# Parameterized Synthesis for Fragments of First-Order Logic Over Data Words

**DOI:** 10.1007/978-3-030-45231-5_6

**Published:** 2020-04-17

**Authors:** Béatrice Bérard, Benedikt Bollig, Mathieu Lehaut, Nathalie Sznajder

**Affiliations:** 8 ENS/CNRS, Université Paris-Saclay, Cachan, France; 9 University of Duisburg-Essen, Duisburg, Germany; 10 Sorbonne Université, CNRS, LIP6, 75005 Paris, France; 11 CNRS, LSV & ENS Paris-Saclay, Université Paris-Saclay, Cachan, France

## Abstract

We study the synthesis problem for systems with a parameterized number of processes. As in the classical case due to Church, the system selects actions depending on the program run so far, with the aim of fulfilling a given specification. The difficulty is that, at the same time, the environment executes actions that the system cannot control. In contrast to the case of fixed, finite alphabets, here we consider the case of parameterized alphabets. An alphabet reflects the number of processes, which is static but unknown. The synthesis problem then asks whether there is a finite number of processes for which the system can satisfy the specification. This variant is already undecidable for very limited logics. Therefore, we consider a first-order logic without the order on word positions. We show that even in this restricted case synthesis is undecidable if both the system and the environment have access to all processes. On the other hand, we prove that the problem is decidable if the environment only has access to a bounded number of processes. In that case, there is even a cutoff meaning that it is enough to examine a bounded number of process architectures to solve the synthesis problem.

## 1 Introduction

Synthesis deals with the problem of automatically generating a program that satisfies a given specification. The problem goes back to Church [9], who formulated it as follows: The environment and the system alternately select an input symbol and an output symbol from a finite alphabet, respectively, and in this way generate an infinite sequence. The question now is whether the system has a *winning strategy*, which guarantees that the resulting infinite run is contained in a given ($\omega$)-regular language representing the specification, no matter how the environment behaves. This problem is decidable and very well understood [8,37], and it has been extended in several different ways (e.g., [24,26,28,36,43]).

In this paper, we consider a variant of the synthesis problem that allows us to model programs with a variable number of processes. As we then deal with an unbounded number of process identifiers, a fixed finite alphabet is not suitable anymore. It is more appropriate to use an infinite alphabet, in which every

* Partly supported by ANR FREDDA (ANR-17-CE40-0013).

letter contains a process identifier and a program action. One can distinguish two cases here. In [16], a potentially infinite number of data values are involved in an infinite program run (e.g. by dynamic process generation). In a *parameterized* system [4, 13], on the other hand, one has an unknown but *static* number of processes so that, along each run, the number of processes is finite. In this paper, we are interested in the latter, i.e., parameterized case. Parameterized programs are ubiquitous and occur, e.g., in distributed algorithms, ad-hoc networks, telecommunication protocols, cache-coherence protocols, swarm robotics, and biological systems. The synthesis question asks whether the system has a winning strategy for some number of processes (existential version) or no matter how many processes there are (universal version).

Over infinite alphabets, there are a variety of different specification languages (e.g., [5, 11, 12, 19, 29, 33, 40]). Unlike in the case of finite alphabets, there is no canonical definition of regular languages. In fact, the synthesis problem has been studied for N-memory automata [7], the Logic of Repeating Values [16], and register automata [15,30,31]. Though there is no agreement on a "regular" automata model, first-order (FO) logic over data words can be considered as a canonical logic, and this is the specification language we consider here. In addition to classical FO logic on words over finite alphabets, it provides a predicate $x \sim y$ to express that two events $x$ and $y$ are triggered by the same process. Its two-variable fragment $\mathrm{FO}^2$ has a decidable emptiness and universality problem [5] and is, therefore, a promising candidate for the synthesis problem.

Previous generalizations of Church's synthesis problem to infinite alphabets were generally *synchronous* in the sense that the system and the environment perform their actions in strictly alternating order. This assumption was made, e.g., in the above-mentioned recent papers [7, 15, 16, 30, 31]. If there are several processes, however, it is realistic to relax this condition, which leads us to an *asynchronous* setting in which the system has no influence on when the environment acts. Like in [21], where the asynchronous case for a fixed number of processes was considered, we only make the reasonable fairness assumption that the system is not blocked forever.

In summary, the synthesis problem over infinite alphabets can be classified as (*i*) parameterized vs. dynamic, (*ii*) synchronous vs. asynchronous, and (*iii*) according to the specification language (register automata, Logic of Repeating Values, FO logic, etc.). As explained above, we consider here the *parameterized asynchronous case for specifications written in FO logic*. To the best of our knowledge, this combination has not been considered before. For flexible modeling, we also distinguish between three types of processes: those that can only be controlled by the system; those that can only be controlled by the environment; and finally those that can be triggered by both. A partition into system and environment processes is also made in [3, 18], but for a fixed number of processes and in the presence of an arena in terms of a Petri net.

Let us briefly describe our results. We show that the general case of the synthesis problem is undecidable for $\mathrm{FO}^2$ logic. This follows from an adaptation of an undecidability result from [16, 17] for a fragment of the Logic of Repeating

Values [11]. We therefore concentrate on an orthogonal logic, namely FO without the order on the word positions. First, we show that this logic can essentially count processes and actions of a given process up to some threshold. Though it has limited expressive power (albeit orthogonal to that of $\mathrm{FO}^2$), it leads to intricate behaviors in the presence of an uncontrollable environment. In fact, we show that the synthesis problem is still undecidable. Due to the lack of the order relation, the proof requires a subtle reduction from the reachability problem in 2-counter Minsky machines. However, it turns out that the synthesis problem is decidable if the number of processes that are controllable by the environment is bounded, while the number of system processes remains unbounded. In this case, there is even a cutoff $k$, an important measure for parameterized systems (cf. [4] for an overview): If the system has a winning strategy for $k$ processes, then it has one for any number of processes greater than $k$, and the same applies to the environment. The proofs of both main results rely on a reduction of the synthesis problem to turn-based *parameterized vector games*, in which, similar to Petri nets, tokens corresponding to processes are moved around between states.

The paper is structured as follows. In Section 2, we define FO logic (especially FO without word order), and in Section 3, we present the parameterized synthesis problem. In Section 4, we transform a given formula into a normal form and finally into a parameterized vector game. Based on this reduction, we investigate cutoff properties and show our (un)decidability results in Section 5. We conclude in Section 6. Some proof details can be found in the long version of this paper [2]

## 2 Preliminaries

For a finite or infinite alphabet $\Sigma$, let $\Sigma^*$ and $\Sigma^\omega$ denote the sets of finite and, respectively, infinite words over $\Sigma$. The empty word is $\varepsilon$. Given $w \in \Sigma^* \cup \Sigma^\omega$, let $|w|$ denote the length of $w$ and $Pos(w)$ its set of positions: $|w| = n$ and $Pos(w) = \{1, \ldots, n\}$ if $w = \sigma_1\sigma_2 \ldots \sigma_n \in \Sigma^*$, and $|w| = \omega$ and $Pos(w) = \{1, 2, \ldots\}$ if $w \in \Sigma^\omega$. Let $w[i]$ be the $i$-th letter of $w$ for all $i \in Pos(w)$.

**Executions.** We consider programs involving a finite (but not fixed) number of processes. Processes are controlled by antagonistic protagonists, System and Environment. Accordingly, each process has a *type* among $\mathbb{T} = \{\mathsf{s}, \mathsf{e}, \mathsf{se}\}$, and we let $\mathbb{P}_\mathsf{s}$, $\mathbb{P}_\mathsf{e}$, and $\mathbb{P}_\mathsf{se}$ denote the pairwise disjoint finite sets of processes controlled by System, by Environment, and by both System and Environment, respectively. We let $\mathbb{P}$ denote the triple $(\mathbb{P}_\mathsf{s}, \mathbb{P}_\mathsf{e}, \mathbb{P}_\mathsf{se})$. Abusing notation, we sometimes refer to $\mathbb{P}$ as the disjoint union $\mathbb{P}_\mathsf{s} \cup \mathbb{P}_\mathsf{e} \cup \mathbb{P}_\mathsf{se}$.

Given any set $S$, vectors $s \in S^\mathbb{T}$ are usually referred to as triples $s = (s_\mathsf{s}, s_\mathsf{e}, s_\mathsf{se})$. Moreover, for $s, s' \in \mathbb{N}^\mathbb{T}$, we write $s \leq s'$ if $s_\theta \leq s'_\theta$ for all $\theta \in \mathbb{T}$. Finally, let $s + s' = (s_\mathsf{s} + s'_\mathsf{s},\ s_\mathsf{e} + s'_\mathsf{e},\ s_\mathsf{se} + s'_\mathsf{se})$.

Processes can execute actions from a finite alphabet $A$. Whenever an action is executed, we would like to know whether it was triggered by System or by Environment. Therefore, $A$ is partitioned into $A = A_\mathsf{s} \uplus A_\mathsf{e}$. Let $\Sigma_\mathsf{s} = A_\mathsf{s} \times (\mathbb{P}_\mathsf{s} \cup \mathbb{P}_\mathsf{se})$ and $\Sigma_\mathsf{e} = A_\mathsf{e} \times (\mathbb{P}_\mathsf{e} \cup \mathbb{P}_\mathsf{se})$. Their union $\Sigma = \Sigma_\mathsf{s} \cup \Sigma_\mathsf{e}$ is the set of *events*. A word $w \in \Sigma^* \cup \Sigma^\omega$ is called a $\mathbb{P}$-*execution*.

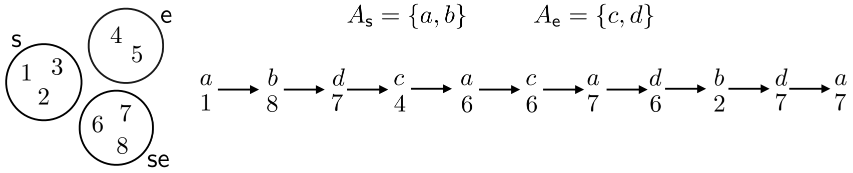

**Fig. 1.** Representation of $\mathbb{P}$-execution as a mathematical structure

**Logic.** Formulas of our logic are evaluated over $\mathbb{P}$-executions. We fix an infinite supply $\mathcal{V} = \{x, y, z, \ldots\}$ of variables, which are interpreted as processes from $\mathbb{P}$ or positions of the execution. The logic $\mathrm{FO}_A[\sim, <, +1]$ is given by the grammar

$$\varphi \ ::= \ \theta(x) \mid a(x) \mid x = y \mid x \sim y \mid x < y \mid +1(x, y) \mid \neg\varphi \mid \varphi \vee \varphi \mid \exists x.\varphi$$

where $x, y \in \mathcal{V}$, $\theta \in \mathbb{T}$, and $a \in A$. Conjunction ($\wedge$), universal quantification ($\forall$), implication ($\Longrightarrow$), *true*, and *false* are obtained as abbreviations as usual.

Let $\varphi \in \mathrm{FO}_A[\sim, <, +1]$. By $\mathit{Free}(\varphi) \subseteq \mathcal{V}$, we denote the set of variables that occur free in $\varphi$. If $\mathit{Free}(\varphi) = \emptyset$, then we call $\varphi$ a *sentence*. We sometimes write $\varphi(x_1, \ldots, x_n)$ to emphasize the fact that $\mathit{Free}(\varphi) \subseteq \{x_1, \ldots, x_n\}$.

To evaluate $\varphi$ over a $\mathbb{P}$-execution $w = (a_1, p_1)(a_2, p_2) \ldots$, we consider $(\mathbb{P}, w)$ as a structure $\mathcal{S}_{(\mathbb{P},w)} = (\mathbb{P} \uplus \mathit{Pos}(w), \mathbb{P}_\mathsf{s}, \mathbb{P}_\mathsf{e}, \mathbb{P}_\mathsf{se}, (R_a)_{a \in A}, \sim, <, +1)$ where $\mathbb{P} \uplus \mathit{Pos}(w)$ is the universe, $\mathbb{P}_\mathsf{s}$ $\mathbb{P}_\mathsf{e}$, and $\mathbb{P}_\mathsf{se}$ are interpreted as unary relations, $R_a$ is the unary relation $\{i \in \mathit{Pos}(w) \mid a_i = a\}$, $< \, = \{(i, j) \in \mathit{Pos}(w) \times \mathit{Pos}(w) \mid i < j\}$, $+1 = \{(i, i + 1) \mid 1 \leq i < |w|\}$, and $\sim$ is the smallest equivalence relation over $\mathbb{P} \uplus \mathit{Pos}(w)$ containing

- $(p, i)$ for all $p \in \mathbb{P}$ and $i \in \mathit{Pos}(w)$ such that $p = p_i$, and
- $(i, j)$ for all $(i, j) \in \mathit{Pos}(w) \times \mathit{Pos}(w)$ such that $p_i = p_j$.

An equivalence class of $\sim$ is often simply referred to as a *class*. Note that it contains exactly one process.

*Example 1.* Suppose $A_\mathsf{s} = \{a, b\}$ and $A_\mathsf{e} = \{c, d\}$. Let the set of processes $\mathbb{P}$ be given by $\mathbb{P}_\mathsf{s} = \{1, 2, 3\}$, $\mathbb{P}_\mathsf{e} = \{4, 5\}$, and $\mathbb{P}_\mathsf{se} = \{6, 7, 8\}$. Moreover, let $w = (a, 1)(b, 8)(d, 7)(c, 4)(a, 6)(c, 6)(a, 7)(d, 6)(b, 2)(d, 7)(a, 7) \in \Sigma^*$. Figure 1 illustrates $\mathcal{S}_{(\mathbb{P},w)}$. The edge relation represents $+1$, its transitive closure is $<$. $\lhd$

An *interpretation* for $(\mathbb{P}, w)$ is a partial mapping $I : \mathcal{V} \to \mathbb{P} \cup \mathit{Pos}(w)$. Suppose $\varphi \in \mathrm{FO}_A[\sim, <, +1]$ such that $\mathit{Free}(\varphi) \subseteq \mathrm{dom}(I)$. The satisfaction relation $(\mathbb{P}, w), I \models \varphi$ is then defined as expected, based on the structure $\mathcal{S}_{(\mathbb{P},w)}$ and interpreting free variables according to $I$. For example, let $w = (a_1, p_1)(a_2, p_2) \ldots$ and $i \in \mathit{Pos}(w)$. Then, for $I(x) = i$, we have $(\mathbb{P}, w), I \models a(x)$ if $a_i = a$.

We identify some fragments of $\mathrm{FO}_A[\sim, <, +1]$. For $R \subseteq \{\sim, <, +1\}$, let $\mathrm{FO}_A[R]$ denote the set of formulas that do not use symbols in $\{\sim, <, +1\} \setminus R$. Moreover, $\mathrm{FO}^2_A[R]$ denotes the fragment of $\mathrm{FO}_A[R]$ that uses only two (reusable) variables.

Let $\varphi(x_1,\ldots,x_n,y) \in \mathrm{FO}_A[\sim,<,+1]$ be a formula and $m \in \mathbb{N}$. We use $\exists^{\geq m} y.\varphi(x_1,\ldots,x_n,y)$ as an abbreviation for

$$\exists y_1 \ldots \exists y_m. \bigwedge_{1\leq i<j\leq m} \neg(y_i = y_j) \wedge \bigwedge_{1\leq i\leq m} \varphi(x_1,\ldots,x_n,y_i)\,,$$

if $m > 0$, and $\exists^{\geq 0} y.\varphi(x_1,\ldots,x_n,y) = \mathit{true}$. Thus, $\exists^{\geq m} y.\varphi$ says that there are at least $m$ distinct elements that verify $\varphi$. We also use $\exists^{=m} y.\varphi$ as an abbreviation for $\exists^{\geq m} y.\varphi \wedge \neg\exists^{\geq m+1} y.\varphi$. Note that $\varphi \in \mathrm{FO}_A[R]$ implies that $\exists^{\geq m} y.\varphi \in \mathrm{FO}_A[R]$ and $\exists^{=m} y.\varphi \in \mathrm{FO}_A[R]$.

*Example 2.* Let $A$, $\mathbb{P}$, and $w$ be like in Example 1 and Figure 1.

- $\varphi_1 = \forall x.\big((\mathsf{s}(x) \vee \mathsf{se}(x)) \implies \exists y.(x \sim y \wedge (a(y) \vee b(y)))\big)$ says that each process that System can control executes at least one system action. We have $\varphi_1 \in \mathrm{FO}^2_A[\sim]$ and $(\mathbb{P},w) \not\models \varphi_1$, as process 3 is idle.
- $\varphi_2 = \forall x.\big(d(x) \implies \exists y.(x \sim y \wedge a(y))\big)$ says that, for every $d$, there is an $a$ on the same process. We have $\varphi_2 \in \mathrm{FO}^2_A[\sim]$ and $(\mathbb{P},w) \models \varphi_2$.
- $\varphi_3 = \forall x.\big(d(x) \implies \exists y.(x \sim y \wedge x < y \wedge a(y))\big)$ says that every $d$ is *eventually* followed by an $a$ executed by the same process. We have $\varphi_3 \in \mathrm{FO}^2_A[\sim,<]$ and $(\mathbb{P},w) \not\models \varphi_3$: The event $(d,6)$ is not followed by some $(a,6)$.
- $\varphi_4 = \forall x.\big((\exists^{=2} y.(x \sim y \wedge a(y))) \iff (\exists^{=2} y.(x \sim y \wedge d(y)))\big)$ says that each class contains exactly two occurrences of $a$ iff it contains exactly two occurrences of $d$. Moreover, $\varphi_4 \in \mathrm{FO}_A[\sim]$ and $(\mathbb{P},w) \models \varphi_4$. Note that $\varphi_4 \notin \mathrm{FO}^2_A[\sim]$, as $\exists^{=2} y$ requires the use of three different variable names. ◁

## 3 Parameterized Synthesis Problem

We define an asynchronous synthesis problem. A $\mathbb{P}$-*strategy* (for System) is a mapping $f : \Sigma^* \to \Sigma_{\mathsf{s}} \cup \{\varepsilon\}$. A $\mathbb{P}$-execution $w = \sigma_1\sigma_2\ldots \in \Sigma^* \cup \Sigma^\omega$ is $f$-*compatible* if, for all $i \in \mathit{Pos}(w)$ such that $\sigma_i \in \Sigma_{\mathsf{s}}$, we have $f(\sigma_1\ldots\sigma_{i-1}) = \sigma_i$. We call $w$ $f$-*fair* if the following hold: $(i)$ If $w$ is finite, then $f(w) = \varepsilon$, and $(ii)$ if $w$ is infinite and $f(\sigma_1\ldots\sigma_{i-1}) \neq \varepsilon$ for infinitely many $i \geq 1$, then $\sigma_j \in \Sigma_{\mathsf{s}}$ for infinitely many $j \geq 1$.

Let $\varphi \in \mathrm{FO}_A[\sim,<,+1]$ be a sentence. We say that $f$ is $\mathbb{P}$-*winning* for $\varphi$ if, for every $\mathbb{P}$-execution $w$ that is $f$-compatible and $f$-fair, we have $(\mathbb{P},w) \models \varphi$.

The existence of a $\mathbb{P}$-strategy that is $\mathbb{P}$-winning for a given formula does not depend on the concrete process identities but only on the cardinality of the sets $\mathbb{P}_{\mathsf{s}}$, $\mathbb{P}_{\mathsf{e}}$, and $\mathbb{P}_{\mathsf{se}}$. This motivates the following definition of winning triples for a formula. Given $\varphi$, let $\mathit{Win}(\varphi)$ be the set of triples $(k_{\mathsf{s}},k_{\mathsf{e}},k_{\mathsf{se}}) \in \mathbb{N}^{\mathbb{T}}$ for which there is $\mathbb{P} = (\mathbb{P}_{\mathsf{s}},\mathbb{P}_{\mathsf{e}},\mathbb{P}_{\mathsf{se}})$ such that $|\mathbb{P}_\theta| = k_\theta$ for all $\theta \in \mathbb{T}$ and there is a $\mathbb{P}$-strategy that is $\mathbb{P}$-winning for $\varphi$.

Let $\mathbb{0} = \{0\}$ and $k_{\mathsf{e}}, k_{\mathsf{se}} \in \mathbb{N}$. In this paper, we focus on the intersection of $\mathit{Win}(\varphi)$ with the sets $\mathbb{N} \times \mathbb{0} \times \mathbb{0}$ (which corresponds to the usual satisfiability problem); $\mathbb{N} \times \{k_{\mathsf{e}}\} \times \{k_{\mathsf{se}}\}$ (there is a constant number of environment and mixed processes); $\mathbb{N} \times \mathbb{N} \times \{k_{\mathsf{se}}\}$ (there is a constant number of mixed processes); $\mathbb{0} \times \mathbb{0} \times \mathbb{N}$ (each process is controlled by both System and Environment).

**Definition 3 (synthesis problem).** *For fixed* $\mathfrak{F} \in \{\mathrm{FO}, \mathrm{FO}^2\}$*, set of relation symbols* $R \subseteq \{\sim, <, +1\}$*, and* $\mathcal{N}_{\mathsf{s}}, \mathcal{N}_{\mathsf{e}}, \mathcal{N}_{\mathsf{se}} \subseteq \mathbb{N}$*, the (parameterized) synthesis problem is given as follows:*

| $\textsc{Synth}(\mathfrak{F}[R], \mathcal{N}_{\mathsf{s}}, \mathcal{N}_{\mathsf{e}}, \mathcal{N}_{\mathsf{se}})$ | |
|---|---|
| **Input:** | $A = A_{\mathsf{s}} \uplus A_{\mathsf{e}}$ *and a sentence* $\varphi \in \mathfrak{F}_A[R]$ |
| **Question:** | $\mathit{Win}(\varphi) \cap (\mathcal{N}_{\mathsf{s}} \times \mathcal{N}_{\mathsf{e}} \times \mathcal{N}_{\mathsf{se}}) \neq \emptyset$ ? |

*The* satisfiability problem *for* $\mathfrak{F}[R]$ *is defined as* $\textsc{Synth}(\mathfrak{F}[R], \mathbb{N}, \mathbb{0}, \mathbb{0})$.

*Example 4.* Suppose $A_{\mathsf{s}} = \{a, b\}$ and $A_{\mathsf{e}} = \{c, d\}$, and consider the formulas $\varphi_1$–$\varphi_4$ from Example 2.

First, we have $\mathit{Win}(\varphi_1) = \mathbb{N}^{\mathbb{T}}$. Given an arbitrary $\mathbb{P}$ and any total order $\sqsubseteq$ over $\mathbb{P}_{\mathsf{s}} \cup \mathbb{P}_{\mathsf{se}}$, a possible $\mathbb{P}$-strategy $f$ that is $\mathbb{P}$-winning for $\varphi_1$ maps $w \in \Sigma^*$ to $(a, p)$ if $p$ is the smallest process from $\mathbb{P}_{\mathsf{s}} \cup \mathbb{P}_{\mathsf{se}}$ wrt. $\sqsubseteq$ that does not occur in $w$, and that returns $\varepsilon$ for $w$ if all processes from $\mathbb{P}_{\mathsf{s}} \cup \mathbb{P}_{\mathsf{se}}$ already occur in $w$.

For the three formulas $\varphi_2$, $\varphi_3$, and $\varphi_4$, observe that, since $d$ is an environment action, if there is at least one process that is exclusively controlled by Environment, then there is no winning strategy. Hence we must have $\mathbb{P}_{\mathsf{e}} = \emptyset$. In fact, this condition is sufficient in the three cases and the strategies described below show that all three sets $\mathit{Win}(\varphi_2)$, $\mathit{Win}(\varphi_3)$, and $\mathit{Win}(\varphi_4)$ are equal to $\mathbb{N} \times \mathbb{0} \times \mathbb{N}$.

- For $\varphi_2$, the very same strategy as for $\varphi_1$ also works in this case, producing an $a$ for every process in $\mathbb{P}_{\mathsf{s}} \cup \mathbb{P}_{\mathsf{se}}$, whether there is a $d$ or not.
- For $\varphi_3$, a winning strategy $f$ will apply the previous mechanism iteratively, performing $(a, p)$ for $p \in \mathbb{P}_{\mathsf{se}} = \{p_0, \ldots, p_{n-1}\}$ over and over again: $f(w) = (a, p_i)$ where $i$ is the number of occurrences of letters from $\Sigma_{\mathsf{s}}$ modulo $n$. By the fairness assumption, this guarantees satisfaction of $\varphi_3$. A more "economical" winning strategy $f'$ may organize pending requests in terms of $d$ in a queue and acknowledge them successively. More precisely, given $u \in \mathbb{P}^*$ and $\sigma \in \Sigma$, we define another word $u \odot \sigma \in \mathbb{P}^*$ by $u \odot (d, p) = u \cdot p$ (inserting $p$ in the queue) and $(p \cdot u) \odot (a, p) = u$ (deleting it). In all other cases, $u \odot \sigma = u$. Let $w = \sigma_1 \ldots \sigma_n \in \Sigma^*$, with queue $((\varepsilon \odot \sigma_1) \odot \sigma_2 \ldots) \odot \sigma_n = p_1 \ldots p_k$. We let $f'(w) = \varepsilon$ if $k = 0$, and $f'(w) = (a, p_1)$ if $k \geq 1$.
- For $\varphi_4$, the strategy $f'$ for $\varphi_3$ ensures that every $d$ has a *corresponding* $a$ so that, in the long run, there are as many $a$'s as $d$'s in every class. ◁

Another interesting question is whether System (or Environment) has a winning strategy as soon as the number of processes is big enough. This leads to the notion of a cutoff (cf. [4] for an overview): Let $\mathcal{N}_{\mathsf{s}}, \mathcal{N}_{\mathsf{e}}, \mathcal{N}_{\mathsf{se}} \subseteq \mathbb{N}$ and $W \subseteq \mathbb{N}^{\mathbb{T}}$. We call $\boldsymbol{k_0} \in \mathbb{N}^{\mathbb{T}}$ a *cutoff* of $W$ wrt. $(\mathcal{N}_{\mathsf{s}}, \mathcal{N}_{\mathsf{e}}, \mathcal{N}_{\mathsf{se}})$ if $\boldsymbol{k_0} \in \mathcal{N}_{\mathsf{s}} \times \mathcal{N}_{\mathsf{e}} \times \mathcal{N}_{\mathsf{se}}$ and either

- for all $\boldsymbol{k} \in \mathcal{N}_{\mathsf{s}} \times \mathcal{N}_{\mathsf{e}} \times \mathcal{N}_{\mathsf{se}}$ such that $\boldsymbol{k} \geq \boldsymbol{k_0}$, we have $\boldsymbol{k} \in W$, or
- for all $\boldsymbol{k} \in \mathcal{N}_{\mathsf{s}} \times \mathcal{N}_{\mathsf{e}} \times \mathcal{N}_{\mathsf{se}}$ such that $\boldsymbol{k} \geq \boldsymbol{k_0}$, we have $\boldsymbol{k} \notin W$.

Let $\mathfrak{F} \in \{\mathrm{FO}, \mathrm{FO}^2\}$ and $R \subseteq \{\sim, <, +1\}$. If, for every alphabet $A = A_{\mathsf{s}} \uplus A_{\mathsf{e}}$ and every sentence $\varphi \in \mathfrak{F}_A[R]$, the set $\mathit{Win}(\varphi)$ has a computable cutoff wrt.

**Table 1.** Summary of results. Our contributions are highlighted in **bold**.

| Synthesis | $(\mathbb{N}, \mathbb{0}, \mathbb{0})$ | $(\mathbb{N}, \{k_{\mathsf{e}}\}, \{k_{\mathsf{se}}\})$ | $(\mathbb{N}, \mathbb{N}, \mathbb{0})$ | $(\mathbb{0}, \mathbb{0}, \mathbb{N})$ |
|---|---|---|---|---|
| $\text{FO}^2[\sim, <, +1]$ | decidable [5] | ? | ? | **undecidable** |
| $\text{FO}^2[\sim, <]$ | NEXPTIME-c. [5] | ? | ? | ? |
| $\text{FO}[\sim]$ | **decidable** | **decidable** | ?* | **undecidable** |

*We show, however, that there is no cutoff.

$(\mathcal{N}_{\mathsf{s}}, \mathcal{N}_{\mathsf{e}}, \mathcal{N}_{\mathsf{se}})$, then we know that $\text{Synth}(\mathfrak{F}[R], \mathcal{N}_{\mathsf{s}}, \mathcal{N}_{\mathsf{e}}, \mathcal{N}_{\mathsf{se}})$ is decidable, as it can be reduced to a finite number of simple synthesis problems over a finite alphabet. The latter can be solved, e.g., using attractor-based backward search (cf. [42]). This is how we will show decidability of $\text{Synth}(\text{FO}[\sim], \mathbb{N}, \{k_{\mathsf{e}}\}, \{k_{\mathsf{se}}\})$ for all $k_{\mathsf{e}}, k_{\mathsf{se}} \in \mathbb{N}$.

Our contributions are summarized in Table 1. Note that known satisfiability results for data logic apply to our logic, as processes can be simulated by treating every $\theta \in \mathbb{T}$ as an ordinary letter. Let us first state undecidability of the general synthesis problem, which motivates the study of other FO fragments.

**Theorem 5.** *The problem* $\text{Synth}(\text{FO}^2[\sim, <, +1], \mathbb{0}, \mathbb{0}, \mathbb{N})$ *is undecidable.*

*Proof (sketch).* We adapt the proof from [16, 17] reducing the halting problem for 2-counter machines. We show that their encoding can be expressed in our logic, even if we restrict it to two variables, and can also be adapted to the asynchronous setting. □

## 4 FO[∼] and Parameterized Vector Games

Due to the undecidability result of Theorem 5, one has to switch to other fragments of first-order logic. We will henceforth focus on the logic $\text{FO}[\sim]$ and establish some important properties, such as a normal form, that will allow us to deduce a couple of results, both positive and negative.

### 4.1 Satisfiability and Normal Form for FO[∼]

We first show that $\text{FO}[\sim]$ logic essentially allows one to count letters in a class up to some threshold, and to count such classes up to some other threshold. Let $B \in \mathbb{N}$ and $\ell \in \{0, \ldots, B\}^A$. Intuitively, $\ell(a)$ imposes a constraint on the number of occurrences of $a$ in a class. We first define an $\text{FO}_A[\sim]$-formula $\psi_{B,\ell}(y)$ verifying that, in the class defined by $y$, the number of occurrences of each letter $a \in A$, counted up to $B$, is $\ell(a)$:

$$\psi_{B,\ell}(y) \;=\; \bigwedge_{\substack{a \in A \,\mid \\ \ell(a) < B}} \exists^{=\ell(a)} z.\big(y \sim z \wedge a(z)\big) \wedge \bigwedge_{\substack{a \in A \,\mid \\ \ell(a) = B}} \exists^{\geq \ell(a)} z.\big(y \sim z \wedge a(z)\big)$$

**Theorem 6 (normal form for FO[$\sim$]).** *Let $\varphi \in \mathrm{FO}_A[\sim]$ be a sentence. There is a computable $B \in \mathbb{N}$ such that $\varphi$ is effectively equivalent to a disjunction of conjunctions of formulas of the form $\exists^{\bowtie m} y.\big(\theta(y) \wedge \psi_{B,\ell}(y)\big)$ where $\bowtie \in \{\geq, =\}$, $m \in \mathbb{N}$, $\theta \in \mathbb{T}$, and $\ell \in \{0, \ldots, B\}^A$.*

The normal form can be obtained using known normal-form constructions [23,41] for general FO logic [2], or using Ehrenfeucht-Fraïssé games [39], or using a direct inductive transformation in the spirit of [23].

*Example 7.* Recall the formula $\varphi_4 = \forall x.\big((\exists^{=2} y.(x \sim y \wedge a(y))) \iff (\exists^{=2} y.(x \sim y \wedge d(y)))\big) \in \mathrm{FO}_A[\sim]$ from Example 2, over $A_\mathsf{s} = \{a, b\}$ and $A_\mathsf{e} = \{c, d\}$. An equivalent formula in normal form is $\varphi_4' = \bigwedge_{\theta \in \mathbb{T},\ \ell \in Z} \exists^{=0} y.\big(\theta(y) \wedge \psi_{3,\ell}(y)\big)$ where $Z$ is the set of vectors $\ell \in \{0, \ldots, 3\}^A$ such that $\ell(a) = 2 \neq \ell(d)$ or $\ell(d) = 2 \neq \ell(a)$. The formula indeed says that there is no class with $=2$ occurrences of $a$ and $\neq 2$ occurrences of $d$ or vice versa, which is equivalent to $\varphi_4$. ◁

Thanks to the normal form, it is sufficient to test finitely many structures to determine whether a given formula is satisfiable:

**Corollary 8.** *The satisfiability problem for* FO[$\sim$] *over data words is decidable. Moreover, every satisfiable* $\mathrm{FO}_A[\sim]$ *formula has a finite model.*

Note that the satisfiability problem for $\mathrm{FO}^2[\sim]$ is already NEXPTIME-hard, due to NEXPTIME-hardness for two-variable logic with unary relations only [14, 20,22]. In fact, it is NEXPTIME-complete due to the upper bound for $\mathrm{FO}^2[\sim, <]$ [5]. It is worth mentioning that two-variable logic with one equivalence relation on arbitrary structures also has the finite-model property [32].

### 4.2 From Synthesis to Parameterized Vector Games

Exploiting the normal form for $\mathrm{FO}_A[\sim]$, we now present a reduction of the synthesis problem to a strictly turn-based two-player game. This game is conceptually simpler and easier to reason about. The reduction works in both directions, which will allow us to derive both decidability and undecidability results.

Note that, given a formula $\varphi \in \mathrm{FO}_A[\sim]$ (which we suppose to be in normal form with threshold $B$), the order of letters in an execution does not matter. Thus, given some $\mathbb{P}$, a reasonable strategy for Environment would be to just "wait and see". More precisely, it does not put Environment into a worse position if, given the current execution $w \in \Sigma^*$, it lets the System execute as many actions as it wants in terms of a word $u \in \Sigma_\mathsf{s}^*$. Due to the fairness assumption, System would be able to execute all the letters from $u$ anyway. Environment can even require System to play a word $u$ such that $(\mathbb{P}, wu) \models \varphi$. If System is not able to produce such a word, Environment can just sit back and do nothing. Conversely, upon $wu$ satisfying $\varphi$, Environment has to be able to come up with a word $v \in \Sigma_\mathsf{e}^*$ such that $(\mathbb{P}, wuv) \not\models \varphi$. This leads to a turn-based game in which System and Environment play in strictly alternate order and have to provide a satisfying and, respectively, falsifying execution.

In a second step, we can get rid of process identifiers: According to our normal form, all we are interested in is the *number* of processes that agree on their letters counted up to threshold $B$. That is, a finite execution can be abstracted as a *configuration* $C : L \to \mathbb{N}^{\mathbb{T}}$ where $L = \{0, \dots, B\}^A$. For $\ell \in L$ and $C(\ell) = (n_{\mathsf{s}}, n_{\mathsf{e}}, n_{\mathsf{se}})$, $n_\theta$ is the number of processes of type $\theta$ whose letter count up to threshold $B$ corresponds to $\ell$. We can also say that $\ell$ contains $n_\theta$ tokens of type $\theta$. If it is System's turn, it will pick some pairs $(\ell, \ell')$ and move some tokens of type $\theta \in \{\mathsf{s}, \mathsf{se}\}$ from $\ell$ to $\ell'$, provided $\ell(a) \leq \ell'(a)$ for all $a \in A_{\mathsf{s}}$ and $\ell(a) = \ell'(a)$ for all $a \in A_{\mathsf{e}}$. This actually corresponds to adding more system letters in the corresponding processes. The Environment proceeds analogously.

Finally, the formula $\varphi$ naturally translates to an acceptance condition $\mathcal{F} \subseteq \mathfrak{C}^L$ over configurations, where $\mathfrak{C}$ is the set of *local acceptance conditions*, which are of the form $(\bowtie_{\mathsf{s}} n_{\mathsf{s}}\,,\ \bowtie_{\mathsf{e}} n_{\mathsf{e}}\,,\ \bowtie_{\mathsf{se}} n_{\mathsf{se}})$ where $\bowtie_{\mathsf{s}}, \bowtie_{\mathsf{e}}, \bowtie_{\mathsf{se}} \in \{=, \geq\}$ and $n_{\mathsf{s}}, n_{\mathsf{e}}, n_{\mathsf{se}} \in \mathbb{N}$.

We end up with a turn-based game in which, similarly to a VASS game [1,6,10,27,38], System and Environment move tokens along vectors from $L$. Note that, however, our games have a very particular structure so that undecidability for VASS games does not carry over to our setting. Moreover, existing decidability results do not allow us to infer our cutoff results below.

In the following, we will formalize *parameterized vector games.*

**Definition 9.** *A* parameterized vector game *(or simply* game*) is given by a triple* $\mathcal{G} = (A, B, \mathcal{F})$ *where* $A = A_{\mathsf{s}} \uplus A_{\mathsf{e}}$ *is the finite alphabet,* $B \in \mathbb{N}$ *is a bound, and, letting* $L = \{0, \dots, B\}^A$*,* $\mathcal{F} \subseteq \mathfrak{C}^L$ *is a finite set called* acceptance condition*.*

*Locations.* Let $\ell_0$ be the location such that $\ell_0(a) = 0$ for all $a \in A$. For $\ell \in L$ and $a \in A$, we define $\ell + a$ by $(\ell + a)(b) = \ell(b)$ for $b \neq a$ and $(\ell + a)(b) = \max\{\ell(a) + 1, B\}$ otherwise. This is extended for all $u \in A^*$ and $a \in A$ by $\ell + \varepsilon = \ell$ and $\ell + ua = (\ell + u) + a$. By $\langle\!\langle w \rangle\!\rangle$, we denote the location $\ell_0 + w$.

*Configurations.* As explained above, a *configuration* of $\mathcal{G}$ is a mapping $C : L \to \mathbb{N}^{\mathbb{T}}$. Suppose that, for $\ell \in L$ and $\theta \in \mathbb{T}$, we have $C(\ell) = (n_{\mathsf{s}}, n_{\mathsf{e}}, n_{\mathsf{se}})$. Then, we let $C(\ell, \theta)$ refer to $n_\theta$. By *Conf*, we denote the set of all configurations.

*Transitions.* A *system transition* (respectively *environment transition*) is a mapping $\tau : L \times L \to (\mathbb{N} \times \{0\} \times \mathbb{N})$ (respectively $\tau : L \times L \to (\{0\} \times \mathbb{N} \times \mathbb{N})$) such that, for all $(\ell, \ell') \in L \times L$ with $\tau(\ell, \ell') \neq (0, 0, 0)$, there is a word $w \in A_{\mathsf{s}}^*$ (respectively $w \in A_{\mathsf{e}}^*$) such that $\ell' = \ell + w$. Let $T_{\mathsf{s}}$ denote the set of system transitions, $T_{\mathsf{e}}$ the set of environment transitions, and $T = T_{\mathsf{s}} \cup T_{\mathsf{e}}$ the set of all transitions.

For $\tau \in T$, let the mappings $out_\tau, in_\tau : L \to \mathbb{N}^{\mathbb{T}}$ be defined by $out_\tau(\ell) = \sum_{\ell' \in L} \tau(\ell, \ell')$ and $in_\tau(\ell) = \sum_{\ell' \in L} \tau(\ell', \ell)$ (recall that sum is component-wise). We say that $\tau \in T$ is *applicable* at $C \in Conf$ if, for all $\ell \in L$, we have $out_\tau(\ell) \leq C(\ell)$ (component-wise). Abusing notation, we let $\tau(C)$ denote the configuration $C'$ defined by $C'(\ell) = C(\ell) - out_\tau(\ell) + in_\tau(\ell)$ for all $\ell \in L$. Moreover, for $\tau(\ell, \ell') = (n_{\mathsf{s}}, n_{\mathsf{e}}, n_{\mathsf{se}})$ and $\theta \in \mathbb{T}$, we let $\tau(\ell, \ell', \theta)$ refer to $n_\theta$.

*Plays.* Let $C \in Conf$. We write $C \models \mathcal{F}$ if there is $\kappa \in \mathcal{F}$ such that, for all $\ell \in L$, we have $C(\ell) \models \kappa(\ell)$ (in the expected manner). A $C$-*play*, or simply *play*, is a finite sequence $\pi = C_0 \tau_1 C_1 \tau_2 C_2 \dots \tau_n C_n$ alternating between configurations and transitions (with $n \geq 0$) such that $C_0 = C$ and, for all $i \in \{1, \dots, n\}$, $C_i = \tau_i(C_{i-1})$ and

- if $i$ is odd, then $\tau_i \in T_{\mathsf{s}}$ and $C_i \models \mathcal{F}$ (System's move),
- if $i$ is even, then $\tau_i \in T_{\mathsf{e}}$ and $C_i \not\models \mathcal{F}$ (Environment's move).

The set of all $C$-plays is denoted by $\mathit{Plays}_C$.

*Strategies.* A *$C$-strategy* for System is a partial mapping $f : \mathit{Plays}_C \to T_{\mathsf{s}}$ such that $f(C)$ is defined and, for all $\pi = C_0\tau_1C_1\dots\tau_iC_i \in \mathit{Plays}_C$ with $\tau = f(\pi)$ defined, we have that $\tau$ is applicable at $C_i$ and $\tau(C_i) \models \mathcal{F}$. Play $\pi = C_0\tau_1C_1\dots\tau_nC_n$ is

- *$f$-compatible* if, for all odd $i \in \{1, \dots, n\}$, $\tau_i = f(C_0\tau_1C_1\dots\tau_{i-1}C_{i-1})$,
- *$f$-maximal* if it is not the strict prefix of an $f$-compatible play,
- *winning* if $C_n \models \mathcal{F}$.

We say that $f$ is *winning* for System (from $C$) if all $f$-compatible $f$-maximal $C$-plays are winning. Finally, $C$ is *winning* if there is a $C$-strategy that is winning. Note that, given an initial configuration $C$, we deal with an acyclic finite reachability game so that, if there is a winning $C$-strategy, then there is a positional one, which only depends on the last configuration.

For $\boldsymbol{k} \in \mathbb{N}^{\mathbb{T}}$, let $C_{\boldsymbol{k}}$ denote the configuration that maps $\ell_0$ to $\boldsymbol{k}$ and all other locations to $(0, 0, 0)$. We set $\mathit{Win}(\mathcal{G}) = \{\boldsymbol{k} \in \mathbb{N}^{\mathbb{T}} \mid C_{\boldsymbol{k}} \text{ is winning for System}\}$.

**Definition 10 (game problem).** *For sets $\mathcal{N}_{\mathsf{s}}, \mathcal{N}_{\mathsf{e}}, \mathcal{N}_{\mathsf{se}} \subseteq \mathbb{N}$, the game problem is given as follows:*

| $\textsc{Game}(\mathcal{N}_{\mathsf{s}}, \mathcal{N}_{\mathsf{e}}, \mathcal{N}_{\mathsf{se}})$ | |
|---|---|
| **Input:** | *Parameterized vector game $\mathcal{G}$* |
| **Question:** | $\mathit{Win}(\mathcal{G}) \cap (\mathcal{N}_{\mathsf{s}} \times \mathcal{N}_{\mathsf{e}} \times \mathcal{N}_{\mathsf{se}}) \neq \emptyset$ ? |

One can show that parameterized vector games are equivalent to the synthesis problem in the following sense:

**Lemma 11.** *For every sentence $\varphi \in \mathrm{FO}_A[\sim]$, there is a parameterized vector game $\mathcal{G} = (A, B, \mathcal{F})$ such that $\mathit{Win}(\varphi) = \mathit{Win}(\mathcal{G})$. Conversely, for every parameterized vector game $\mathcal{G} = (A, B, \mathcal{F})$, there is a sentence $\varphi \in \mathrm{FO}_A[\sim]$ such that $\mathit{Win}(\mathcal{G}) = \mathit{Win}(\varphi)$. Both directions are effective.*

*Example 12.* To illustrate parameterized vector games and the reduction from the synthesis problem, consider the formula $\varphi_4' = \bigwedge_{\theta \in \mathbb{T},\, \ell \in Z} \exists^{=0} y.\big(\theta(y) \wedge \psi_{3,\ell}(y)\big)$ in normal form from Example 7. For simplicity, we assume that $A_{\mathsf{s}} = \{a\}$ and $A_{\mathsf{e}} = \{d\}$. That is, $Z$ is the set of vectors $\langle\!\langle a^i d^j \rangle\!\rangle \in L = \{0, \dots, 3\}^{\{a,d\}}$ such that $i = 2 \neq j$ or $j = 2 \neq i$. Figure 2 illustrates a couple of configurations $C_0, \dots, C_5 : L \to \mathbb{N}^{\mathbb{T}}$. The leftmost location in a configuration is $\ell_0$, the rightmost

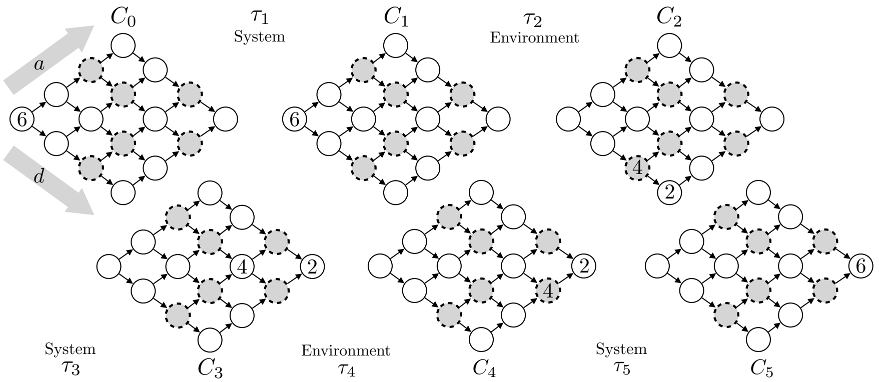

**Fig. 2.** A play of a parameterized vector game location $\langle\!\langle a^3d^3\rangle\!\rangle$, the topmost one $\langle\!\langle a^3\rangle\!\rangle$, and the one at the bottom $\langle\!\langle d^3\rangle\!\rangle$. Self-loops have been omitted, and locations from $Z$ have gray background and a dashed border.

Towards an equivalent game $\mathcal{G} = (A, 3, \mathcal{F})$, it remains to determine the acceptance condition $\mathcal{F}$. Recall that $\varphi'_4$ says that every class contains two occurrences of $a$ iff it contains two occurrences of $d$. This is reflected by the acceptance condition $\mathcal{F} = \{\kappa\}$ where $\kappa(\ell) = ({=}0\,, {=}0\,, {=}0)$ for all $\ell \in Z$ and $\kappa(\ell) = ({\geq}0\,, {\geq}0\,, {\geq}0)$ for all $\ell \in L \setminus Z$. With this, a configuration is accepting iff no token is on a location from $Z$ (a gray location).

We can verify that $Win(\mathcal{G}) = Win(\varphi'_4) = \mathbb{N} \times \mathbb{0} \times \mathbb{N}$. In $\mathcal{G}$, a uniform winning strategy $f$ for System that works for all $\mathbb{P}$ with $\mathbb{P}_{\mathsf{e}} = \emptyset$ proceeds as follows: System first awaits an Environment's move and then moves each token upwards as many locations as Environment has moved it downwards. Figure 2 illustrates an $f$-maximal $C_{(6,0,0)}$-play that is winning for System. We note that $f$ is a "compressed" version of the winning strategy presented in Example 4, as System makes her moves only when really needed. $\lhd$

## 5 Results for FO[∼] via Parameterized Vector Games

In this section, we present our results for the synthesis problem for FO[∼], which we obtain showing corresponding results for parameterized vector games. In particular, we show that $(\text{FO}[\sim], \mathbb{0}, \mathbb{0}, \mathbb{N})$ and $(\text{FO}[\sim], \mathbb{N}, \mathbb{N}, \mathbb{0})$ do not have a cutoff, whereas $(\text{FO}[\sim], \mathbb{N}, \{k_{\mathsf{e}}\}, \{k_{\mathsf{se}}\})$ has a cutoff for all $k_{\mathsf{e}}, k_{\mathsf{se}} \in \mathbb{N}$. Finally, we prove that $\textsc{Synth}(\text{FO}[\sim], \mathbb{0}, \mathbb{0}, \mathbb{N})$ is, in fact, undecidable.

**Lemma 13.** *There is a game $\mathcal{G} = (A, B, \mathcal{F})$ such that $Win(\mathcal{G})$ does not have a cutoff wrt. $(\mathbb{0}, \mathbb{0}, \mathbb{N})$.*

*Proof.* We let $A_{\mathsf{s}} = \{a\}$ and $A_{\mathsf{e}} = \{b\}$, as well as $B = 2$. For $k \in \{0, 1, 2\}$, define the local acceptance conditions ${}^{=}k = ({=}0\,, {=}0\,, {=}k)$ and ${}^{\geq}k = ({=}0\,, {=}0\,, {\geq}k)$. Set

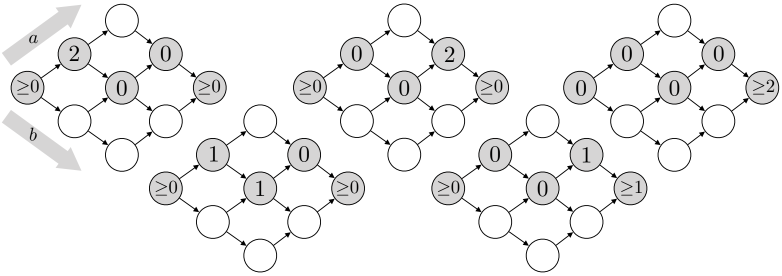

**Fig. 3.** Acceptance conditions for a game with no cutoff wrt. $(\mathbb{0}, \mathbb{0}, \mathbb{N})$

$\ell_1 = \langle\!\langle a \rangle\!\rangle, \ell_2 = \langle\!\langle ab \rangle\!\rangle, \ell_3 = \langle\!\langle a^2 b \rangle\!\rangle$, and $\ell_4 = \langle\!\langle a^2 b^2 \rangle\!\rangle$. For $k_0, \ldots, k_4 \in \{0, 1, 2\}$ and $\bowtie_0, \ldots, \bowtie_4 \in \{=, \geq\}$, let $[{}^{\bowtie_0} k_0\,, {}^{\bowtie_1} k_1\,, {}^{\bowtie_2} k_2\,, {}^{\bowtie_3} k_3\,, {}^{\bowtie_4} k_4]$ denote $\kappa \in \mathfrak{C}^L$ where $\kappa(\ell_i) = ({}^{\bowtie_i} k_i)$ for all $i \in \{0, \ldots, 4\}$ and $\kappa(\ell') = ({}^{=}0)$ for $\ell' \notin \{\ell_0, \ldots, \ell_4\}$. Finally,

$$\mathcal{F} = \left\{ \begin{matrix} [{}^{\geq}0\,, {}^{=}2\,, {}^{=}0\,, {}^{=}0\,, {}^{\geq}0] & [{}^{\geq}0\,, {}^{=}0\,, {}^{=}0\,, {}^{=}2\,, {}^{\geq}0] & [{}^{=}0\,, {}^{=}0\,, {}^{=}0\,, {}^{=}0\,, {}^{\geq}2] \\ [{}^{\geq}0\,, {}^{=}1\,, {}^{=}1\,, {}^{=}0\,, {}^{\geq}0] & [{}^{\geq}0\,, {}^{=}0\,, {}^{=}0\,, {}^{=}1\,, {}^{\geq}1] & \end{matrix} \right\} \cup K_{\mathsf{e}}$$

where $K_{\mathsf{e}} = \{\kappa_\ell \mid \ell \in L$ such that $\ell(b) > \ell(a)\}$ with $\kappa_\ell(\ell') = ({}^{\geq}1)$ if $\ell' = \ell$, and $\kappa_\ell(\ell') = ({}^{\geq}0)$ otherwise. This is illustrated in Figure 3.

There is a winning strategy for System from any initial configuration of size $2n$: Move two tokens from $\ell_0$ to $\ell_1$, wait until Environment sends them both to $\ell_2$, then move them to $\ell_3$, wait until they are moved to $\ell_4$, then repeat with two new tokens from $\ell_0$ until all the tokens are removed from $\ell_0$, and Environment cannot escape $\mathcal{F}$ anymore. However, one can check that there is no winning strategy for initial configurations of odd size. □

**Lemma 14.** *There is a game $\mathcal{G} = (A, B, \mathcal{F})$ such that $Win(\mathcal{G})$ does not have a cutoff wrt.* $(\mathbb{N}, \mathbb{N}, \mathbb{0})$.

*Proof.* We define $\mathcal{G}$ such that System wins only if she has at least as many processes as Environment. Let $A_{\mathsf{s}} = \{a\}$, $A_{\mathsf{e}} = \{b\}$, and $B = 2$. As there are no shared processes, we can safely ignore locations with a letter from both System and Environment. We set $\mathcal{F} = \{\kappa_1, \kappa_2, \kappa_3, \kappa_4\}$ where

$$\begin{matrix} \kappa_1(\langle\!\langle a \rangle\!\rangle) = (=1\,, =0\,, =0) & \kappa_2(\langle\!\langle a \rangle\!\rangle) = (=1\,, =0\,, =0) & \kappa_3(\langle\!\langle a \rangle\!\rangle) = (=0\,, =0\,, =0) \\ \kappa_1(\langle\!\langle b \rangle\!\rangle) = (=0\,, =0\,, =0) & \kappa_2(\langle\!\langle b \rangle\!\rangle) = (=0\,, \geq 2\,, =0) & \kappa_3(\langle\!\langle b \rangle\!\rangle) = (=0\,, \geq 1\,, =0)\,, \end{matrix}$$

$\kappa_4(\ell_0) = (=0\,, =0\,, =0)$, and $\kappa_i(\ell') = (\geq 0\,, \geq 0\,, =0)$ for all other $\ell' \in L$ and $i \in \{1, 2, 3, 4\}$. □

We now turn to the case where the number of processes that can be triggered by Environment is bounded. Note that similar restrictions are imposed in other settings to get decidability, such as limiting the environment to a finite (Boolean) domain [16] or restricting to one environment process [3, 18]. We obtain decidability of the synthesis problem via a cutoff construction:

**Theorem 15.** *Given $k_{\mathsf{e}}, k_{\mathsf{se}} \in \mathbb{N}$, every game $\mathcal{G} = (A, B, \mathcal{F})$ has a cutoff wrt.* $(\mathbb{N}, \{k_{\mathsf{e}}\}, \{k_{\mathsf{se}}\})$. *More precisely: Let $K$ be the largest constant that occurs in $\mathcal{F}$. Moreover, let $Max = (k_{\mathsf{e}} + k_{\mathsf{se}}) \cdot |A_{\mathsf{e}}| \cdot B$ and $\hat{N} = |L|^{Max+1} \cdot K$. Then, $(\hat{N}, k_{\mathsf{e}}, k_{\mathsf{se}})$ is a cutoff of $Win(\mathcal{G})$ wrt.* $(\mathbb{N}, \{k_{\mathsf{e}}\}, \{k_{\mathsf{se}}\})$.

*Proof.* We will show that, for all $N \geq \hat{N}$,

$$(N, k_{\mathsf{e}}, k_{\mathsf{se}}) \in Win(\mathcal{G}) \iff (N+1, k_{\mathsf{e}}, k_{\mathsf{se}}) \in Win(\mathcal{G})\,.$$

The main observation is that, when $C$ contains more than $K$ tokens in a given $\ell \in L$, adding more tokens in $\ell$ will not change whether $C \models \mathcal{F}$. Given $C, C' \in Conf$, we write $C <_{\mathsf{e}} C$ if $C \neq C'$ and there is $\tau \in T_{\mathsf{e}}$ such that $\tau(C) = C'$. Note that the length $d$ of a chain $C_0 <_{\mathsf{e}} C_1 <_{\mathsf{e}} \ldots <_{\mathsf{e}} C_d$ is bounded by $Max$. In other words, $Max$ is the maximal number of transitions that Environment can do in a play. For all $d \in \{0, \ldots, Max\}$, let $Conf_d$ be the set of configurations $C \in Conf$ such that the longest chain in $(Conf, <_{\mathsf{e}})$ starting from $C$ has length $d$.

*Claim.* Suppose that $C \in Conf_d$ and $\ell \in L$ such that $C(\ell) = (N, n_{\mathsf{e}}, n_{\mathsf{se}})$ with $N \geq |L|^{d+1} \cdot K$ and $n_{\mathsf{e}}, n_{\mathsf{se}} \in \mathbb{N}$. Set $D = C[\ell \mapsto (N+1, n_{\mathsf{e}}, n_{\mathsf{se}})]$. Then,

$$C \text{ is winning for System} \iff D \text{ is winning for System.}$$

To show the claim, we proceed by induction on $d \in \mathbb{N}$, which is illustrated in Figure 4. In each implication, we distinguish the cases $d = 0$ and $d \geq 1$. For the latter, we assume that equivalence holds for all values strictly smaller than $d$.

For $\tau \in T_{\mathsf{s}}$ and $\ell, \ell' \in L$, we let $\tau[(\ell, \ell', \mathsf{s})\text{++}]$ denote the transition $\eta \in T_{\mathsf{s}}$ given by $\eta(\ell_1, \ell_2, \mathsf{e}) = \tau(\ell_1, \ell_2, \mathsf{e}) = 0$, $\eta(\ell_1, \ell_2, \mathsf{se}) = \tau(\ell_1, \ell_2, \mathsf{se})$, $\eta(\ell_1, \ell_2, \mathsf{s}) = \tau(\ell_1, \ell_2, \mathsf{s}) + 1$ if $(\ell_1, \ell_2) = (\ell, \ell')$, and $\eta(\ell_1, \ell_2, \mathsf{s}) = \tau(\ell_1, \ell_2, \mathsf{s})$ if $(\ell_1, \ell_2) \neq (\ell, \ell')$. We define $\tau[(\ell, \ell', \mathsf{s})\text{−−}]$ similarly (provided $\tau(\ell, \ell', \mathsf{s}) \geq 1$).

$\Longrightarrow$: Let $f$ be a winning strategy for System from $C \in Conf_d$. Let $\tau' = f(C)$ and $C' = \tau'(C)$. Note that $C' \models \mathcal{F}$. Since $C(\ell, \mathsf{s}) = N \geq |L|^{d+1} \cdot K$, there is $\ell' \in L$ such that $\ell + w = \ell'$ for some $w \in A_{\mathsf{s}}^*$ and $C'(\ell', \mathsf{s}) = N' \geq |L|^d \cdot K$.

We show that $D = C[\ell \mapsto (N+1, n_{\mathsf{e}}, n_{\mathsf{se}})]$ is winning for System by exhibiting a corresponding winning strategy $g$ from $D$ that will carefully control the position of the additional token. First, set $g(D) = \eta'$ where $\eta' = \tau'[(\ell, \ell', \mathsf{s})\text{++}]$. Let $D' = \eta'(D)$. We obtain $D'(\ell', \mathsf{s}) = N' + 1$. Note that, since $N' \geq K$, the acceptance condition $\mathcal{F}$ cannot distinguish between $C'$ and $D'$. Thus, we have $D' \models \mathcal{F}$.

Case $d = 0$: As, for all transitions $\eta'' \in T_{\mathsf{e}}$, we have $\eta''(D') = D' \models \mathcal{F}$, we reached a maximal play that is winning for System. We deduce that $D$ is winning for System.

Case $d \geq 1$: Take any $\eta'' \in T_{\mathsf{e}}$ and $D''$ such that $D'' = \eta''(D') \not\models \mathcal{F}$. Let $\tau'' = \eta''$ and $C'' = \tau''(C')$. Note that $D'' = C''[(\ell', \mathsf{s}) \mapsto N+1]$, $C'' = D''[(\ell', \mathsf{s}) \mapsto N]$, and $C'', D'' \in Conf_{d^-}$ for some $d^- < d$. As $f$ is a winning strategy for System from $C$, we have that $C''$ is winning for System. By induction hypothesis, $D''$ is winning for System, say by winning strategy $g''$. We let $g(D\,\eta'\,D'\,\eta''\,\pi) = g''(\pi)$ for all $D''$-plays $\pi$. For all unspecified plays, let $g$ return any applicable system transition. Altogether, for any choice of $\eta''$, we have that $g''$ is winning from $D''$. Thus, $g$ is a winning strategy from $D$.

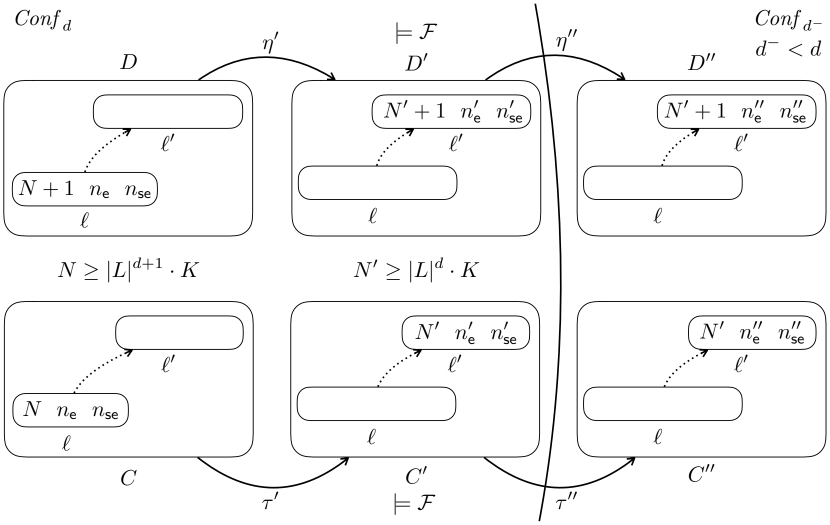

**Fig. 4.** Induction step in the cutoff construction

$\Longleftarrow$: Suppose $g$ is a winning strategy for System from $D$. Thus, for $\eta' = g(D)$ and $D' = \eta'(D)$, we have $D' \models \mathcal{F}$. Recall that $D(\ell, \mathsf{s}) \geq (|L|^{d+1} \cdot K) + 1$. We distinguish two cases:

1. Suppose there is $\ell' \in L$ such that $\ell \neq \ell'$, $D'(\ell', \mathsf{s}) = N' + 1$ for some $N' \geq |L|^d \cdot K$, and $\eta'(\ell, \ell', \mathsf{s}) \geq 1$. Then, we set $\tau' = \eta'[(\ell, \ell', \mathsf{s})\text{--}]$.
2. Otherwise, we have $D'(\ell, \mathsf{s}) \geq (|L|^d \cdot K) + 1$, and we set $\tau' = \eta'$ (as well as $\ell' = \ell$ and $N' = N$).

Let $C' = \tau'(C)$. Since $D' \models \mathcal{F}$, one obtains $C' \models \mathcal{F}$.

Case $d = 0$: For all transitions $\tau'' \in T_{\mathsf{e}}$, we have $\tau''(C') = C' \models \mathcal{F}$. Thus, we reached a maximal play that is winning for System. We deduce that $C$ is winning for System.

Case $d \geq 1$: Take any $\tau'' \in T_{\mathsf{e}}$ such that $C'' = \tau''(C') \not\models \mathcal{F}$. Let $\eta'' = \tau''$ and $D'' = \eta''(D')$. We have $C'' = D''[(\ell', \mathsf{s}) \mapsto N']$, $D'' = C''[(\ell', \mathsf{s}) \mapsto N' + 1]$, and $C'', D'' \in Conf_{d^-}$ for some $d^- < d$. As $D''$ is winning for System, by induction hypothesis, $C''$ is winning for System, say by winning strategy $f''$. We let $f(C\,\tau'\,C'\,\tau''\,\pi) = f''(\pi)$ for all $C''$-plays $\pi$. For all unspecified plays, let $f$ return an arbitrary applicable system transition. Again, for any choice of $\tau''$, $f''$ is winning from $C''$. Thus, $f$ is a winning strategy from $C$.

This conclude the proof of the claim and, therefore, of Theorem 15. $\square$

**Corollary 16.** *Let $k_{\mathsf{e}}, k_{\mathsf{se}} \in \mathbb{N}$ be the number of environment and the number of mixed processes, respectively. The problems* Game$(\mathbb{N}, \{k_{\mathsf{e}}\}, \{k_{\mathsf{se}}\})$ *and* Synth(FO[$\sim$]$, \mathbb{N}, \{k_{\mathsf{e}}\}, \{k_{\mathsf{se}}\})$ *are decidable.*

In particular, by Theorem 15, the game problem can be reduced to an exponential number of acyclic finite-state games whose size (and hence the time complexity for determining the winner) is exponential in the cutoff and, therefore, doubly exponential in the size of the alphabet, the bound $B$, and the fixed number of processes that are controllable by the environment.

**Theorem 17.** $\text{Game}(\mathbb{0}, \mathbb{0}, \mathbb{N})$ *and* $\text{Synth}(\text{FO}[\sim], \mathbb{0}, \mathbb{0}, \mathbb{N})$ *are undecidable.*

*Proof.* We provide a reduction from the halting problem for 2-counter machines (2CM) to $\text{Game}(\mathbb{0}, \mathbb{0}, \mathbb{N})$. A 2CM $M = (Q, \Delta, \mathsf{c}_1, \mathsf{c}_2, q_0, q_h)$ has two counters, $\mathsf{c}_1$ and $\mathsf{c}_2$, a finite set of states $Q$, and a set of transitions $\Delta \subseteq Q \times \mathsf{Op} \times Q$ where $\mathsf{Op} = \{\mathsf{c}_i{+}{+}, \mathsf{c}_i{-}{-}, \mathsf{c}_i{=}{=}0 \mid i \in \{1,2\}\}$. Moreover, we have an initial state $q_0 \in Q$ and a halting state $q_h \in Q$. A configuration of $M$ is a triple $\gamma = (q, \nu_1, \nu_2) \in Q \times \mathbb{N} \times \mathbb{N}$ giving the current state and the current respective counter values. The initial configuration is $\gamma_0 = (q_0, 0, 0)$ and the set of halting configurations is $F = \{q_h\} \times \mathbb{N} \times \mathbb{N}$. For $t \in \Delta$, configuration $(q', \nu_1', \nu_2')$ is a ($t$-)successor of $(q, \nu_1, \nu_2)$, written $(q, \nu_1, \nu_2) \vdash_t (q', \nu_1', \nu_2')$, if there is $i \in \{1,2\}$ such that $\nu_{3-i}' = \nu_{3-i}$ and one of the following holds: $(i)$ $t = (q, \mathsf{c}_i{+}{+}, q')$ and $\nu_i' = \nu_i + 1$, or $(ii)$ $t = (q, \mathsf{c}_i{-}{-}, q')$ and $\nu_i' = \nu_i - 1$, or $(iii)$ $t = (q, \mathsf{c}_i{=}{=}0, q')$ and $\nu_i = \nu_i' = 0$. A run of $M$ is a (finite or infinite) sequence $\gamma_0 \vdash_{t_1} \gamma_1 \vdash_{t_2} \ldots$. The 2CM halting problem asks whether there is a run reaching a configuration in $F$. It is known to be undecidable [34].

We fix a 2CM $M = (Q, \Delta, \mathsf{c}_1, \mathsf{c}_2, q_0, q_h)$. Let $A_\mathsf{s} = Q \cup \Delta \cup \{a_1, a_2\}$ and $A_\mathsf{e} = \{b\}$ with $a_1$, $a_2$, and $b$ three fresh symbols. We consider the game $\mathcal{G} = (A, B, \mathcal{F})$ with $A = A_\mathsf{s} \uplus A_\mathsf{e}$, $B = 4$, and $\mathcal{F}$ defined below. Let $L = \{0, \ldots, B\}^A$. Since there are only processes shared by System and Environment, we alleviate notation and consider that a configuration is simply a mapping $C : L \to \mathbb{N}$. From now on, to avoid confusion, we refer to configurations of the 2CM $M$ as $M$-configurations, and to configurations of $\mathcal{G}$ as $\mathcal{G}$-configurations.

Intuitively, every valid run of $M$ will be encoded as a play in $\mathcal{G}$, and the acceptance condition will enforce that, if a player in $\mathcal{G}$ deviates from a valid play, then she will lose immediately. At any point in the play, there will be at most one process with only a letter from $Q$ played, which will represent the current state in the simulated 2CM run. Similarly, there will be at most one process with only a letter from $\Delta$ to represent what transition will be taken next. Finally, the value of counter $\mathsf{c}_i$ will be encoded by the number of processes with exactly two occurrences of $a_i$ and two occurrences of $b$ (i.e., $C(\langle\!\langle a_i^2 b^2 \rangle\!\rangle)$).

To increase counter $\mathsf{c}_i$, the players will move a new token to $\langle\!\langle a_i^2 b^2 \rangle\!\rangle$, and to decrease it, they will move, together, a token from $\langle\!\langle a_i^2 b^2 \rangle\!\rangle$ to $\langle\!\langle a_i^4 b^4 \rangle\!\rangle$. Observe that, if $\mathsf{c}_i$ has value 0, then $C(\langle\!\langle a_i^2 b^2 \rangle\!\rangle) = 0$ in the corresponding configuration of the game. As expected, it is then impossible to simulate the decrement of $\mathsf{c}_i$. Environment's only role is to acknowledge System's actions by playing its (only) letter when System simulates a valid run. If System tries to cheat, she loses immediately.

*Encoding an $M$-configuration.* Let us be more formal. Suppose $\gamma = (q, \nu_1, \nu_2)$ is an $M$-configuration and $C$ a $\mathcal{G}$-configuration. We say that $C$ *encodes* $\gamma$ if

- $C(\langle\!\langle q \rangle\!\rangle) = 1$, $C(\langle\!\langle a_1^2 b^2 \rangle\!\rangle) = \nu_1$, $C(\langle\!\langle a_2^2 b^2 \rangle\!\rangle) = \nu_2$,
- $C(\ell) \geq 0$ for all $\ell \in \{\ell_0\} \cup \{\langle\!\langle \hat{q}^2 b^2 \rangle\!\rangle, \langle\!\langle t^2 b^2 \rangle\!\rangle, \langle\!\langle a_i^4 b^4 \rangle\!\rangle \mid \hat{q} \in Q, t \in \Delta, i \in \{1,2\}\}$,
- $C(\ell) = 0$ for all other $\ell \in L$.

We then write $\gamma = \mathsf{m}(C)$. Let $\mathbb{C}(\gamma)$ be the set of $\mathcal{G}$-configurations $C$ that encode $\gamma$. We say that a $\mathcal{G}$-configuration $C$ is *valid* if $C \in \mathbb{C}(\gamma)$ for some $\gamma$.

*Simulating a transition of $M$.* Let us explain how we go from a $\mathcal{G}$-configuration encoding $\gamma$ to a $\mathcal{G}$-configuration encoding a successor $M$-configuration $\gamma'$. Observe that System cannot change by herself the $M$-configuration encoded. If, for instance, she tries to change the current state $q$, she might move one process from $\ell_0$ to $\langle\!\langle q' \rangle\!\rangle$, but then the $\mathcal{G}$-configuration is not valid anymore. We need to move the process in $\langle\!\langle q \rangle\!\rangle$ into $\langle\!\langle q^2 b^2 \rangle\!\rangle$ and this requires the cooperation of Environment.

Assume that the game is in configuration $C$ encoding $\gamma = (q, \nu_1, \nu_2)$. System will pick a transition $t$ starting in state $q$, say, $t = (q, \mathsf{c}_1{+}{+}, q')$. From configuration $C$, System will go to the configuration $C_1$ defined by $C_1(\langle\!\langle t \rangle\!\rangle) = 1$, $C_1(\langle\!\langle a_1 \rangle\!\rangle) = 1$, and $C_1(\ell) = C(\ell)$ for all other $\ell \in L$.

If the transition $t$ is correctly chosen, Environment will go to a configuration $C_2$ defined by $C_2(\langle\!\langle q \rangle\!\rangle) = 0$, $C_2(\langle\!\langle qb \rangle\!\rangle) = 1$, $C_2(\langle\!\langle t \rangle\!\rangle) = 0$, $C_2(\langle\!\langle tb \rangle\!\rangle) = 1$, $C_2(\langle\!\langle a_1 \rangle\!\rangle) = 0$, $C_2(\langle\!\langle a_1 b \rangle\!\rangle) = 1$ and, for all other $\ell \in L$, $C_2(\ell) = C_1(\ell)$. This means that Environment moves processes in locations $\langle\!\langle t \rangle\!\rangle$, $\langle\!\langle q \rangle\!\rangle$, $\langle\!\langle a_1 \rangle\!\rangle$ to locations $\langle\!\langle tb \rangle\!\rangle$, $\langle\!\langle qb \rangle\!\rangle$, $\langle\!\langle a_1 b \rangle\!\rangle$, respectively.

To finish the transition, System will now move a process to the destination state $q'$ of $t$, and go to configuration $C_3$ defined by $C_3(\langle\!\langle q' \rangle\!\rangle) = 1$, $C_3(\langle\!\langle tb \rangle\!\rangle) = 0$, $C_3(\langle\!\langle t^2 b \rangle\!\rangle) = 1$, $C_3(\langle\!\langle qb \rangle\!\rangle) = 0$, $C_3(\langle\!\langle q^2 b \rangle\!\rangle) = 1$, $C_3(\langle\!\langle a_1 b \rangle\!\rangle) = 0$, $C_3(\langle\!\langle a_1^2 b \rangle\!\rangle) = 1$, and $C_3(\ell) = C_2(\ell)$ for all other $\ell \in L$.

Finally, Environment moves to configuration $C_4$ given by $C_4(\langle\!\langle t^2 b \rangle\!\rangle) = 0$, $C_4(\langle\!\langle t^2 b^2 \rangle\!\rangle) = C_3(\langle\!\langle t^2 b^2 \rangle\!\rangle) + 1$, $C_4(\langle\!\langle q^2 b \rangle\!\rangle) = 0$, $C_4(\langle\!\langle q^2 b^2 \rangle\!\rangle) = C_3(\langle\!\langle q^2 b^2 \rangle\!\rangle) + 1$, $C_4(\langle\!\langle a_1^2 b \rangle\!\rangle) = 0$, $C_4(\langle\!\langle a_1^2 b^2 \rangle\!\rangle) = C_3(\langle\!\langle a_1^2 b^2 \rangle\!\rangle) + 1$, and $C_4(\ell) = C_3(\ell)$ for all other $\ell \in L$. Observe that $C_4 \in \mathbb{C}((q', \nu_1 + 1, \nu_2))$.

Other types of transitions will be simulated similarly. To force System to start the simulation in $\gamma_0$, and not in any $M$-configuration, the configurations $C$ such that $C(\langle\!\langle q_0^2 b^2 \rangle\!\rangle) = 0$ and $C(\langle\!\langle q \rangle\!\rangle) = 1$ for $q \neq q_0$ are not valid, and will be losing for System.

*Acceptance condition.* It remains to define $\mathcal{F}$ in a way that enforces the above sequence of $\mathcal{G}$-configurations. Let $L_{\checkmark} = \{\ell_0\} \cup \{\langle\!\langle a_i^2 b^2 \rangle\!\rangle, \langle\!\langle a_i^4 b^4 \rangle\!\rangle \mid i \in \{1,2\}\} \cup \{\langle\!\langle q^2 b^2 \rangle\!\rangle \mid q \in Q\} \cup \{\langle\!\langle t^2 b^2 \rangle\!\rangle \mid t \in \Delta\}$ be the set of elements in $L$ whose values do not affect the acceptance of the configuration. By $[\ell_1 \bowtie_1 n_1, \ldots, \ell_k \bowtie_k n_k]$, we denote $\kappa \in \mathfrak{C}^L$ such that $\kappa(\ell_i) = (\bowtie_i n_i)$ for $i \in \{1, \ldots, k\}$ and $\kappa(\ell) = (=0)$ for all $\ell \in L \setminus \{\ell_1, \ldots, \ell_k\}$. Moreover, for a set of locations $\hat{L} \subseteq L$, we let $\hat{L} \geq 0$ stand for "$(\ell \geq 0)$ for all $\ell \in \hat{L}$".

First, we force Environment to play only in response to System by making System win as soon as there is a process where Environment has played more letters than System (see Condition (d) in Table 2).

If $\gamma$ is not halting, the configurations in $\mathbb{C}(\gamma)$ will not be winning for System. Hence, System will have to move to win (Condition (a)).

**Table 2.** Acceptance conditions for the game simulating a 2CM

**Requirements for System**

**(a)** For all $t = (q, \mathsf{op}, q') \in Q$:

$$\mathcal{F}_{(q,t)} = \bigcup_{\hat{q}\in Q}\big\{[\langle\!\langle q\rangle\!\rangle = 1, \langle\!\langle t\rangle\!\rangle = 1, \langle\!\langle a_i\rangle\!\rangle = 1, \ \langle\!\langle \hat{q}^2b^2\rangle\!\rangle \geq 1, \ \big(L_\checkmark \setminus \{\langle\!\langle \hat{q}^2b^2\rangle\!\rangle\}\big) \geq 0]\big\} \quad \text{if } \mathsf{op} = \mathsf{c}_i{+}{+}$$

$$\mathcal{F}_{(q,t)} = \bigcup_{\hat{q}\in Q}\big\{[\langle\!\langle q\rangle\!\rangle = 1, \langle\!\langle t\rangle\!\rangle = 1, \langle\!\langle a_i^3b^2\rangle\!\rangle = 1, \langle\!\langle \hat{q}^2b^2\rangle\!\rangle \geq 1, \ \big(L_\checkmark \setminus \{\langle\!\langle \hat{q}^2b^2\rangle\!\rangle\}\big) \geq 0]\big\} \quad \text{if } \mathsf{op} = \mathsf{c}_i{-}{-}$$

$$\mathcal{F}_{(q,t)} = \bigcup_{\hat{q}\in Q}\big\{[\langle\!\langle q\rangle\!\rangle = 1, \langle\!\langle t\rangle\!\rangle = 1, \langle\!\langle a_i^2b^2\rangle\!\rangle = 0, \langle\!\langle \hat{q}^2b^2\rangle\!\rangle \geq 1, \ \big(L_\checkmark \setminus \{\langle\!\langle \hat{q}^2b^2\rangle\!\rangle, \langle\!\langle a_i^2b^2\rangle\!\rangle\}\big) \geq 0]\big\} \quad \text{if } \mathsf{op} = \mathsf{c}_i{=}{=}0$$

**(b)** For all $t = (q_0, \mathsf{op}, q') \in Q$ such that $\mathsf{op} \in \{\mathsf{c}_i{+}{+}, \mathsf{c}_i{=}{=}0\}$:

$$\mathcal{F}_t = \big\{[\langle\!\langle q_0\rangle\!\rangle = 1, \langle\!\langle t\rangle\!\rangle = 1, \langle\!\langle a_i\rangle\!\rangle = 1, \ell_0 \geq 0]\big\} \quad \text{if } \mathsf{op} = \mathsf{c}_i{+}{+}$$

$$\mathcal{F}_t = \big\{[\langle\!\langle q_0\rangle\!\rangle = 1, \langle\!\langle t\rangle\!\rangle = 1, \ell_0 \geq 0]\big\} \quad \text{if } \mathsf{op} = \mathsf{c}_i{=}{=}0$$

**(c)** For all $t = (q, \mathsf{op}, q') \in Q$:

$$\mathcal{F}_{(q,t,q')} = \big\{[\langle\!\langle q^2b\rangle\!\rangle = 1, \langle\!\langle t^2b\rangle\!\rangle = 1, \langle\!\langle a_i^2b\rangle\!\rangle = 1, \ \langle\!\langle q'\rangle\!\rangle = 1, L_\checkmark \geq 0]\big\} \quad \text{if } \mathsf{op} = \mathsf{c}_i{+}{+}$$

$$\mathcal{F}_{(q,t,q')} = \big\{[\langle\!\langle q^2b\rangle\!\rangle = 1, \langle\!\langle t^2b\rangle\!\rangle = 1, \langle\!\langle a_i^4b^3\rangle\!\rangle = 1, \langle\!\langle q'\rangle\!\rangle = 1, L_\checkmark \geq 0]\big\} \quad \text{if } \mathsf{op} = \mathsf{c}_i{-}{-}$$

$$\mathcal{F}_{(q,t,q')} = \big\{[\langle\!\langle q^2b\rangle\!\rangle = 1, \langle\!\langle t^2b\rangle\!\rangle = 1, L_\checkmark \geq 0]\big\} \quad \text{if } \mathsf{op} = \mathsf{c}_i{=}{=}0$$

**Requirements for Environment**

**(d)** Let $L_{\mathsf{s<e}} = \big\{\ell \in L \mid \big(\sum_{\alpha\in A_\mathsf{s}} \ell(\alpha)\big) < \ell(b)\big\}$. For all $\ell \in L_{\mathsf{s<e}}$: $\mathcal{F}_\ell = [\ell \geq 1, (L \setminus \{\ell\}) \geq 0]$

**(e)** For all $t = (q, \mathsf{op}, q') \in Q$:

$$\mathcal{F}^{\mathsf{e}}_{(q,t)} = \left\{\begin{array}{l} [\langle\!\langle qb\rangle\!\rangle = 1, \langle\!\langle t\rangle\!\rangle = 1, \langle\!\langle a_i\rangle\!\rangle = 1, \ L_\checkmark \geq 0], \ [\langle\!\langle q\rangle\!\rangle = 1, \ \langle\!\langle tb\rangle\!\rangle = 1, \ \langle\!\langle a_i\rangle\!\rangle = 1, \ L_\checkmark \geq 0], \\ [\langle\!\langle q\rangle\!\rangle = 1, \ \langle\!\langle t\rangle\!\rangle = 1, \langle\!\langle a_ib\rangle\!\rangle = 1, L_\checkmark \geq 0], \ [\langle\!\langle qb\rangle\!\rangle = 1, \langle\!\langle tb\rangle\!\rangle = 1, \ \langle\!\langle a_i\rangle\!\rangle = 1, \ L_\checkmark \geq 0], \\ [\langle\!\langle qb\rangle\!\rangle = 1, \langle\!\langle t\rangle\!\rangle = 1, \langle\!\langle a_ib\rangle\!\rangle = 1, L_\checkmark \geq 0], \ [\langle\!\langle q\rangle\!\rangle = 1, \ \langle\!\langle tb\rangle\!\rangle = 1, \ \langle\!\langle a_ib\rangle\!\rangle = 1, L_\checkmark \geq 0] \end{array}\right\} \quad \text{if } \mathsf{op} = \mathsf{c}_i{+}{+}$$

$$\mathcal{F}^{\mathsf{e}}_{(q,t)} = \left\{\begin{array}{l} [\langle\!\langle qb\rangle\!\rangle = 1, \langle\!\langle t\rangle\!\rangle = 1, \langle\!\langle a_i^3b^2\rangle\!\rangle = 1, L_\checkmark \geq 0], \ [\langle\!\langle q\rangle\!\rangle = 1, \ \langle\!\langle tb\rangle\!\rangle = 1, \langle\!\langle a_i^3b^2\rangle\!\rangle = 1, L_\checkmark \geq 0], \\ [\langle\!\langle q\rangle\!\rangle = 1, \ \langle\!\langle t\rangle\!\rangle = 1, \langle\!\langle a_i^3b^3\rangle\!\rangle = 1, L_\checkmark \geq 0], \ [\langle\!\langle qb\rangle\!\rangle = 1, \langle\!\langle tb\rangle\!\rangle = 1, \langle\!\langle a_i^3b^2\rangle\!\rangle = 1, L_\checkmark \geq 0], \\ [\langle\!\langle qb\rangle\!\rangle = 1, \langle\!\langle t\rangle\!\rangle = 1, \langle\!\langle a_i^3b^3\rangle\!\rangle = 1, L_\checkmark \geq 0], \ [\langle\!\langle q\rangle\!\rangle = 1, \ \langle\!\langle tb\rangle\!\rangle = 1, \langle\!\langle a_i^3b^3\rangle\!\rangle = 1, L_\checkmark \geq 0] \end{array}\right\} \quad \text{if } \mathsf{op} = \mathsf{c}_i{-}{-}$$

$$\mathcal{F}^{\mathsf{e}}_{(q,t)} = \big\{[\langle\!\langle qb\rangle\!\rangle = 1, \ \langle\!\langle t\rangle\!\rangle = 1, \ L_\checkmark \geq 0], \ [\langle\!\langle q\rangle\!\rangle = 1, \ \langle\!\langle tb\rangle\!\rangle = 1, \ L_\checkmark \geq 0]\big\} \quad \text{if } \mathsf{op} = \mathsf{c}_i{=}{=}0$$

**(f)** For all $t = (q, \mathsf{op}, q') \in Q$:

$$\mathcal{F}^{\mathsf{e}}_{(q,t,q')} = \left\{\begin{array}{l} [\langle\!\langle q'\rangle\!\rangle = 1, \ \langle\!\langle q^2b\rangle\!\rangle = 1, \langle\!\langle t^2b\rangle\!\rangle \geq 0, \langle\!\langle a_i^2b\rangle\!\rangle \geq 0, L_\checkmark \geq 0], \\ [\langle\!\langle q'\rangle\!\rangle = 1, \ \langle\!\langle q^2b\rangle\!\rangle \geq 0, \langle\!\langle t^2b\rangle\!\rangle = 1, \langle\!\langle a_i^2b\rangle\!\rangle \geq 0, L_\checkmark \geq 0], \\ [\langle\!\langle q'\rangle\!\rangle = 1, \ \langle\!\langle q^2b\rangle\!\rangle \geq 0, \langle\!\langle t^2b\rangle\!\rangle \geq 0, \langle\!\langle a_i^2b\rangle\!\rangle = 1, L_\checkmark \geq 0], \\ [\langle\!\langle q'b\rangle\!\rangle = 1, \langle\!\langle q^2b\rangle\!\rangle \geq 0, \langle\!\langle t^2b\rangle\!\rangle \geq 0, \langle\!\langle a_i^2b\rangle\!\rangle \geq 0, L_\checkmark \geq 0] \end{array}\right\} \quad \text{if } \mathsf{op} = \mathsf{c}_i{+}{+}$$

$$\mathcal{F}^{\mathsf{e}}_{(q,t,q')} = \left\{\begin{array}{l} [\langle\!\langle q'\rangle\!\rangle = 1, \ \langle\!\langle q^2b\rangle\!\rangle = 1, \langle\!\langle t^2b\rangle\!\rangle \geq 0, \langle\!\langle a_i^4b^3\rangle\!\rangle \geq 0, L_\checkmark \geq 0], \\ [\langle\!\langle q'\rangle\!\rangle = 1, \ \langle\!\langle q^2b\rangle\!\rangle \geq 0, \langle\!\langle t^2b\rangle\!\rangle = 1, \langle\!\langle a_i^4b^3\rangle\!\rangle \geq 0, L_\checkmark \geq 0], \\ [\langle\!\langle q'\rangle\!\rangle = 1, \ \langle\!\langle q^2b\rangle\!\rangle \geq 0, \langle\!\langle t^2b\rangle\!\rangle \geq 0, \langle\!\langle a_i^4b^3\rangle\!\rangle = 1, L_\checkmark \geq 0], \\ [\langle\!\langle q'b\rangle\!\rangle = 1, \langle\!\langle q^2b\rangle\!\rangle \geq 0, \langle\!\langle t^2b\rangle\!\rangle \geq 0, \langle\!\langle a_i^4b^3\rangle\!\rangle \geq 0, L_\checkmark \geq 0] \end{array}\right\} \quad \text{if } \mathsf{op} = \mathsf{c}_i{-}{-}$$

$$\mathcal{F}^{\mathsf{e}}_{(q,t,q')} = \left\{\begin{array}{l} [\langle\!\langle q'\rangle\!\rangle = 1, \ \langle\!\langle q^2b\rangle\!\rangle = 1, \langle\!\langle t^2b\rangle\!\rangle \geq 0, L_\checkmark \geq 0], \\ [\langle\!\langle q'\rangle\!\rangle = 1, \ \langle\!\langle q^2b\rangle\!\rangle \geq 0, \langle\!\langle t^2b\rangle\!\rangle = 1, L_\checkmark \geq 0], \\ [\langle\!\langle q'b\rangle\!\rangle = 1, \langle\!\langle q^2b\rangle\!\rangle \geq 0, \langle\!\langle t^2b\rangle\!\rangle \geq 0, \langle\!\langle a_i^4b^3\rangle\!\rangle \geq 0, L_\checkmark \geq 0] \end{array}\right\} \quad \text{if } \mathsf{op} = \mathsf{c}_i{=}{=}0$$

The first transition chosen by System must start from the initial state of $M$. This is enforced by Condition (b).

Once System has moved, Environment will move other processes to leave accepting configurations. The only possible move for her is to add $b$ on a process in locations $\langle\!\langle q \rangle\!\rangle$, $\langle\!\langle t \rangle\!\rangle$, and $\langle\!\langle a_i \rangle\!\rangle$, if $t$ is a transition incrementing counter $\mathsf{c}_i$ (respectively $\langle\!\langle a_i^3 b^2 \rangle\!\rangle$ if $t$ is a transition decrementing counter $\mathsf{c}_i$). All other $\mathcal{G}$-configurations accessible by Environment from already defined accepting configurations are winning for System, as established in Condition (e).

System can now encode the successor configuration of $M$, according to the chosen transition, by moving a process to the destination state of the transition (see Condition (c)).

Finally, Environment makes the necessary transitions for the configuration to be a valid $\mathcal{G}$-configuration. If she deviates, System wins (see Condition (f)).

If Environment reaches a configuration in $\mathbb{C}(\gamma)$ for $\gamma \in F$, System can win by moving the process in $\langle\!\langle q_h \rangle\!\rangle$ to $\langle\!\langle q_h^2 \rangle\!\rangle$. From there, all the configurations reachable by Environment are also winning for System:

$$\mathcal{F}_F = \left\{ [\langle\!\langle q_h^2 \rangle\!\rangle = 1, L_\checkmark \geq 0] \;,\; [\langle\!\langle q_h^2 b \rangle\!\rangle = 1, L_\checkmark \geq 0] \;,\; [\langle\!\langle q_h^2 b^2 \rangle\!\rangle = 1, L_\checkmark \geq 0] \right\} .$$

Finally, the acceptance condition is given by

$$\mathcal{F} = \bigcup_{\ell \in L_{\mathsf{s}<\mathsf{e}}} \mathcal{F}_\ell \cup \bigcup_{t=(q_0,\mathsf{op},q')\in\Delta} \mathcal{F}_t \cup \bigcup_{t=(q,\mathsf{op},q')\in\Delta} \left(\mathcal{F}_{(q,t)} \cup \mathcal{F}^{\mathsf{e}}_{(q,t)} \cup \mathcal{F}_{(q,t,q')} \cup \mathcal{F}^{\mathsf{e}}_{(q,t,q')}\right) \cup \mathcal{F}_F .$$

Note that a correct play can end in three different ways: either there is a process in $\langle\!\langle q_h \rangle\!\rangle$ and System moves it to $\langle\!\langle q_h^2 \rangle\!\rangle$, or System has no transition to pick, or there are not enough processes in $\ell_0$ for System to simulate a new transition. Only the first kind is winning for System.

We can show that there is an accepting run in $M$ iff there is some $k$ such that System has a winning $C_{(0,0,k)}$-strategy for $\mathcal{G}$. □

## 6 Conclusion

There are several questions that we left open and that are interesting in their own right due to their fundamental character. Moreover, in the decidable cases, it will be worthwhile to provide tight bounds on cutoffs and the algorithmic complexity of the decision problem. Like in [7,15,16,30,31], our strategies allow the system to have a global view of the whole program run executed so far. However, it is also perfectly natural to consider uniform local strategies where each process only sees its own actions and possibly those that are revealed according to some causal dependencies. This is, e.g., the setting considered in [3,18] for a fixed number of processes and in [25] for parameterized systems over ring architectures.

Moreover, we would like to study a parameterized version of the control problem [35] where, in addition to a specification, a program in terms of an arena is already given but has to be controlled in a way such that the specification is satisfied. Finally, our synthesis results crucially rely on the fact that the number of processes in each execution is finite. It would be interesting to consider the case with potentially infinitely many processes.

## References

[1] P. A. Abdulla, R. Mayr, A. Sangnier, and J. Sproston. Solving parity games on integer vectors. In P. R. D’Argenio and H. C. Melgratti, editors, *CONCUR 2013 -Concurrency Theory - 24th International Conference, CONCUR 2013, Buenos Aires, Argentina, August 27-30, 2013. Proceedings*, volume 8052 of *Lecture Notes in Computer Science*, pages 106–120. Springer, 2013.

[2] B. Bérard, B. Bollig, M. Lehaut, and N. Sznajder. Parameterized synthesis for fragments of first-order logic over data words. *CoRR*, abs/1910.14294, 2019.

[3] R. Beutner, B. Finkbeiner, and J. Hecking-Harbusch. Translating Asynchronous Games for Distributed Synthesis. In W. Fokkink and R. van Glabbeek, editors, *30th International Conference on Concurrency Theory (CONCUR 2019)*, volume *140 of Leibniz International Proceedings in Informatics (LIPIcs)*, pages 26:1–26:16, Dagstuhl, Germany, 2019. Schloss Dagstuhl-Leibniz-Zentrum fuer Informatik

[4] R. Bloem, S. Jacobs, A. Khalimov, I. Konnov, S. Rubin, H. Veith, and J. Widder. *Decidability of Parameterized Verification*. Morgan & Claypool Publishers, 2015.

[5] M. Bojanczyk, C. David, A. Muscholl, T. Schwentick, and L. Segoufin. Two-variable logic on data words. *ACM Trans. Comput. Log.*, 12(4):27, 2011.

[6] T. Brázdil, P. Jancar, and A. Kucera. Reachability games on extended vector addition systems with states. In *ICALP’10*, Part II, volume 6199 of *LNCS*, pages 478–489. Springer, 2010.

[7] B. Brütsch and W. Thomas. Playing games in the Baire space. In *Proc. Cassting Workshop on Games for the Synthesis of Complex Systems and 3rd Int. Workshop on Synthesis of Complex Parameters*, volume 220 of *EPTCS*, pages 13–25, 2016.

[8] J. R. Büchi, and L. H. Landweber. Solving sequential conditions by finite-state strategies. *Transactions of the American Mathematical Society*, 138:295–311, Apr. 1969.

[9] A. Church. Applications of recursive arithmetic to the problem of circuit synthesis. In *Summaries of the Summer Institute of Symbolic Logic - Volume 1*, pages 3–50. Institute for Defense Analyses, 1957.

[10] J. Courtois and S. Schmitz. Alternating vector addition systems with states. In E. Csuhaj-Varjú, M. Dietzfelbinger, and Z. Ésik, editors, *Mathematical Foundations of Computer Science 2014 - 39th International Symposium, MFCS 2014, Budapest, Hungary, August 25-29, 2014. Proceedings, Part I*, volume 8634 of *Lecture Notes in Computer Science*, pages 220–231. Springer, 2014.

[11] S. Demri, D. D’Souza, and R. Gascon. Temporal logics of repeating values. *J. Log. Comput.*, 22(5):1059–1096, 2012.

[12] S. Demri and R. Lazić.LTL with the freeze quantifier and register automata. *ACM Transactions on Computational Logic*, 10(3), 2009.

[13] J. Esparza. Keeping a crowd safe: On the complexity of parameterized verification. In *STACS’14*, volume 25 of *Leibniz International Proceedings in Informatics*, pages 1–10. Leibniz-Zentrum für Informatik, 2014.

[14] K. Etessami, M. Y. Vardi, and T. Wilke. First-order logic with two variables and unary temporal logic. *Inf. Comput.*, 179(2):279–295, 2002.

[15] L. Exibard, E. Filiot, and P.-A. Reynier. Synthesis of Data Word Transducers. In W. Fokkink and R. van Glabbeek, editors, *30th International Conference on Concurrency Theory (CONCUR 2019)*, volume 140 of *Leibniz International Proceedings in Informatics (LIPIcs)*, pages 24:1–24:15, Dagstuhl, Germany, 2019. Schloss Dagstuhl-Leibniz-Zentrum fuer Informatik.

[16] D. Figueira and M. Praveen. Playing with repetitions in data words using energy games. In A. Dawar and E. Grädel, editors, *Proceedings of the 33rd Annual ACM/IEEE Symposium on Logic in Computer Science, LICS 2018, Oxford, UK, July 09–12, 2018*, pages 404–413. ACM, 2018.

[17] D. Figueira and M. Praveen. Playing with repetitions in data words using energy games. arXiv preprint arXiv:1802.07435, 2018.

[18] B. Finkbeiner and E. Olderog. Petri games: Synthesis of distributed systems with causal memory. *Inf. Comput.*, 253:181–203, 2017.

[19] H. Frenkel, O. Grumberg, and S. Sheinvald. An automata-theoretic approach to model-checking systems and specifications over infinite data domains. *J. Autom. Reasoning*, 63(4):1077–1101, 2019.

[20] M. Fürer. The computational complexity of the unconstrained limited domino problem (with implications for logical decision problems). In E. Börger, G. Hasenjaeger, and D. Rödding, editors, *Logic and Machines: Decision Problems and Complexity, Proceedings of the Symposium "Rekursive Kombinatorik" held from May 23–28, 1983 at the Institut für Mathematische Logik und Grundlagenforschung der Universität Münster/Westfalen*, volume 171 of *Lecture Notes in Computer Science*, pages 312–319. Springer, 1983.

[21] P. Gastin and N. Sznajder. Fair synthesis for asynchronous distributed systems. *ACM Transactions on Computational Logic*, 14(2:9), 2013.

[22] E. Grädel, P. G. Kolaitis, and M. Y. Vardi. On the decision problem for two-variable first-order logic. *Bulletin of Symbolic Logic*, 3(1):53–69, 1997.

[23] W. Hanf. Model-theoretic methods in the study of elementary logic. In J. W. Addison, L. Henkin, and A. Tarski, editors, *The Theory of Models*. North-Holland, Amsterdam, 1965.

[24] F. Horn, W. Thomas, N. Wallmeier, and M. Zimmermann. Optimal strategy synthesis for request-response games. *RAIRO - Theor. Inf. and Applic.*, 49(3):179–203, 2015.

[25] S. Jacobs and R. Bloem. Parameterized synthesis. *Logical Methods in Computer Science*, 10(1), 2014.

[26] S. Jacobs, L. Tentrup, and M. Zimmermann. Distributed synthesis for parameterized temporal logics. *Inf. Comput.*, 262(Part):311–328, 2018.

[27] P. Jancar. On reachability-related games on vector addition systems with states. In *RP’15*, volume 9328 of *LNCS*, pages 50–62. Springer, 2015.

[28] M. Jenkins, J. Ouaknine, A. Rabinovich, and J. Worrell. The church synthesis problem with metric. In M. Bezem, editor, *Computer Science Logic, 25th International Workshop / 20th Annual Conference of the EACSL, CSL 2011, September 12–15, 2011, Bergen, Norway, Proceedings*, volume 12 of *LIPIcs*, pages 307–321. Schloss Dagstuhl - Leibniz-Zentrum fuer Informatik, 2011.

[29] M. Kaminski and N. Francez. Finite-memory automata. *Theoretical Computer Science*, 134(2):329–363, 1994.

[30] A. Khalimov and O. Kupferman. Register-Bounded Synthesis. In W. Fokkink and R. van Glabbeek, editors, *30th International Conference on Concurrency Theory (CONCUR 2019)*, volume 140 of *Leibniz International Proceedings in Informatics (LIPIcs)*, pages 25:1–25:16, Dagstuhl, Germany, 2019. Schloss Dagstuhl-Leibniz-Zentrum fuer Informatik.

[31] A. Khalimov, B. Maderbacher, and R. Bloem. Bounded synthesis of register transducers. In S. K. Lahiri and C. Wang, editors, *Automated Technology for Verification and Analysis - 16th International Symposium, ATVA 2018, Los Angeles, CA, USA, October 7-10, 2018, Proceedings*, volume 11138 of *Lecture Notes in Computer Science*, pages 494–510. Springer, 2018.

[32] E. Kieronski and M. Otto. Small substructures and decidability issues for first-order logic with two variables. *J. Symb. Log.*, 77(3):729–765, 2012.

[33] L. Libkin, T. Tan, and D. Vrgoc. Regular expressions for data words. *J. Comput. Syst. Sci.*, 81(7):1278–1297, 2015.

[34] M. L. Minsky. *Computation: Finite and Infinite Machines*. Prentice Hall, Upper Saddle River, NJ, USA, 1967.

[35] A. Muscholl. Automated synthesis of distributed controllers. In M. M. Halldórsson, K. Iwama, N. Kobayashi, and B. Speckmann, editors, *Automata, Languages, and Programming - 42nd International Colloquium, ICALP 2015, Kyoto, Japan, July 6-10, 2015, Proceedings, Part II*, volume 9135 of *Lecture Notes in Computer Science*, pages 11–27. Springer, 2015.

[36] A. Pnueli and R. Rosner. Distributed reactive systems are hard to synthesize. In *31st Annual Symposium on Foundations of Computer Science, St. Louis, Missouri, USA, October 22-24, 1990, Volume II*, pages 746–757. IEEE Computer Society, 1990.

[37] M. O. Rabin. *Automata on infinite objects and Church’s problem*. Number 13 in Regional Conference Series in Mathematics. American Mathematical Soc., 1972.

[38] J. Raskin, M. Samuelides, and L. V. Begin. Games for counting abstractions. *Electr. Notes Theor. Comput. Sci.*, 128(6):69–85, 2005.

[39] A. Sangnier and O. Stietel. Private communication, 2020.

[40] L. Schröder, D. Kozen, S. Milius, and T. Wißmann. Nominal automata with name binding. In J. Esparza and A. S. Murawski, editors, *Foundations of Software Science and Computation Structures - 20th International Conference, FOSSACS 2017, Held as Part of the European Joint Conferences on Theory and Practice of Software, ETAPS 2017, Uppsala, Sweden, April 22-29, 2017, Proceedings*, volume 10203 of *Lecture Notes in Computer Science*, pages 124–142, 2017.

[41] T. Schwentick and K. Barthelmann. Local normal forms for first-order logic with applications to games and automata. In *Annual Symposium on Theoretical Aspects of Computer Science*, pages 444–454. Springer, 1998.

[42] W. Thomas. Church’s problem and a tour through automata theory. In *Pillars of Computer Science, Essays Dedicated to Boris (Boaz) Trakhtenbrot on the Occasion of His 85th Birthday*, volume 4800 of *Lecture Notes in Computer Science*, pages 635–655. Springer, 2008.

[43] Y. Velner and A. Rabinovich. Church synthesis problem for noisy input. In M. Hofmann, editor, *Foundations of Software Science and Computational Structures - 14th International Conference, FOSSACS 2011, Held as Part of the Joint European Conferences on Theory and Practice of Software, ETAPS 2011, Saarbrücken, Germany, March 26-April 3, 2011. Proceedings*, volume 6604 of *Lecture Notes in Computer Science*, pages 275–289. Springer, 2011.

